# Immune recall improves antibody durability and breadth to SARS-CoV-2 variants

**DOI:** 10.1126/sciimmunol.abp8328

**Published:** 2022-05-12

**Authors:** Yuezhou Chen, Pei Tong, Noah Whiteman, Ali Sanjari Moghaddam, Mehrdad Zarghami, Adam Zuiani, Shaghayegh Habibi, Avneesh Gautam, F. Keerti, Caihong Bi, Tianshu Xiao, Yongfei Cai, Bing Chen, Donna Neuberg, Duane R. Wesemann

**Affiliations:** ^1^ Department of Medicine, Division of Allergy and Immunology, Division of Genetics, Brigham and Women’s Hospital, Harvard Medical School, Boston, MA 02115; ^2^ Ragon Institute of MGH, MIT, and Harvard; ^3^ Laboratory of Molecular Medicine, Boston Children’s Hospital, Boston, MA 02115, USA; ^4^ Department of Data Science, Dana-Farber Cancer Institute, Boston, MA 02215

## Abstract

Key features of immune memory are greater and faster antigen-specific antibody responses to repeat infection. In the setting of immune-evading viral evolution, it is important to understand how far antibody memory recognition stretches across viral variants when memory cells are recalled to action by repeat invasions. It is also important to understand how immune recall influences longevity of secreted antibody responses. We analyzed SARS-CoV-2 variant recognition, dynamics of memory B cells and secreted antibody over time after infection, vaccination, and boosting. We find that a two-dose SARS-CoV-2 vaccination regimen given after natural infection generated greater longitudinal antibody stability and induced maximal antibody magnitudes with enhanced breadth across Beta, Gamma, Delta and Omicron variants. A homologous 3^rd^ mRNA vaccine dose in COVID-naïve individuals conferred greater cross-variant evenness of neutralization potency with stability that was equal to the hybrid immunity conferred by infection plus vaccination. Within unvaccinated individuals who recovered from COVID, enhanced antibody stability over time was observed within a subgroup of individuals that recovered more quickly from COVID and harbored significantly more memory B cells cross-reactive to endemic coronaviruses early after infection. These cross-reactive clones map to the conserved S2 region of SARS-CoV-2 spike with higher somatic hypermutation levels and greater target affinity. We conclude that SARS-CoV-2 antigen challenge histories in humans influence not only the speed and magnitude of antibody responses, but also functional cross-variant antibody repertoire composition and longevity.

## Teaser

Immune recall in vaccination and infection confers greater antibody durability and breadth to SARS-CoV-2 variants.

## INTRODUCTION

The devastating effects of the COVID-19 pandemic can be attenuated by mRNA vaccines encoding for SARS-CoV-2 spike (S) (*1*, *2*). However, emergence of mutated variants (*3*) and waning immunity (*4*, *5*) are continuing major threats. Strategies to maximize robust levels of protective antibodies that are stable over time and retain function across mutating variants through vaccination is a vital goal. Understanding the capabilities of the immune system in this regard, and how available vaccines can elicit them is critical both for the ongoing SARS-CoV-2 pandemic as well as for future vaccine strategies more broadly.

Antibodies are B cell-expressed molecules composed of immunoglobulin (Ig) heavy (H) and light (L) chains and are produced in the context of different IgH isotypes (e.g., IgM, IgG, IgA). Antibodies can evolve to better recognize offending pathogens through B cell clonal selection and somatic hypermutation (SHM), and subsequently produce affinity-matured versions of antibodies at greater speed and magnitude upon recall. While greater speed and magnitude are known advantages of recalled immune memory, how recall influences antibody recognition, breadth and durability across mutating variant pathogens is not well defined. After infection or vaccination, IgG antibodies in some instances can be sustained for decades (*6*). Durable antibody responses require coordinated T and B cell interactions within germinal centers (GCs) (*7*, *8*). GC B cells can differentiate into long-lived plasma cells (LLPCs) or memory B cells. Memory B cells can more efficiently differentiate into antibody secreting plasma cells upon subsequent pathogen invasion, but pre-formed pathogen-specific antibodies produced from LLPCs are prophylactically available before repeat pathogen invasion attempts, and thus provide immediate protection. B cells that are activated outside of GCs can also differentiate into memory B cells (*9*) in addition to shorter-lived versions of antibody-secreting cells.

SARS-CoV-2 infection and vaccination elicits neutralizing antibodies targeting the viral spike glycoprotein (S), a homotrimer of precursor polypeptide chains that are cleaved upon maturation into two fragments, S1 and S2. The S1 region contains the receptor binding domain (RBD) and the N-terminal domain (NTD), both targets of antibodies with potent neutralizing capability. The S2 region is generally conserved across coronaviruses and mediates viral membrane fusion required for entry to host cells (*10*–*14*).

Anti-S neutralizing antibodies are key correlates of disease protection (*4*, *15*–*17*). In this context, declining antibodies over time and immune escape highlight the need to understand how cross-variant repertoire breadth and durability are regulated. To this end, we charted virus-specific antibody durability and cross-variant breadth, with analysis to evaluate neutralization breadth per unit function (i.e., evenness of cross-variant neutralization) and examined how these features dynamically change over time after infection, mRNA vaccination. and mRNA vaccination after infection.

Two-dose mRNA vaccination in COVID-naïve individuals induced robust initial virus-specific antibodies that rapidly decayed to the unvaccinated post-infection plateau. Vaccination after infection induced the greatest antibody magnitudes with enhanced longitudinal stability over time. Notably, we find that while cross-SARS-CoV-2 variant neutralization function tends to track with total antibody magnitudes, vaccine regimens differ in the functional constitution. In this regard, infection, and vaccination after infection, conferred greater cross-variant neutralization coverage per unit potency (i.e., greater cross-variant evenness) compared to a two-dose mRNA vaccine regimen in COVID-naïve individuals. However, a homologous 3^rd^ dose in COVID-naïve vaccinees greatly enhanced not only specific antibody magnitudes, but greater functional evenness of recognition across SARS-C0V-2 variants, including Omicron with stability that approximated hybrid immunity conferred by vaccination after infection.

In addition, we found evidence of likely prior endemic coronavirus memory underlying our previously reported sustainer phenotype (*18*), consisting of improved COVID symptom clearance and more durable antibody durability trajectories in COVID-19 convalescent subjects. Collectively, these studies identify an antibody breadth-enhancing feature induced by infection, and a 3^rd^, but not 2^nd^, homologous mRNA vaccine dose. In addition, a durability-enhancing function is conferred by SARS-CoV-2 infection-induced recall of endemic coronavirus memory.

## RESULTS

### Immune recall by vaccination after prior infection confers greater antibody magnitude and stability

To gain insights into how immune recall influences antibody durability, we charted anti-S and anti-RBD IgG dynamics in three longitudinal cohorts: (i) Individuals who have recovered from SARS-CoV-2 infection and followed >200 days without vaccination follow up (Cohort A) (n = 34), (ii) COVID-19 convalescents who, after being followed for >200 days were also followed an additional >200 days after mRNA vaccination (Cohort B) (n = 28), and (iii) COVID-19-naïve individuals that had received mRNA vaccination for >200 days (naïve vaccinees, Cohort C) (n = 18). COVID-19 convalescents (Cohort A and Cohort B (pre-vaccination)) (n=62) were recruited between March 2020 and June 2020 in Boston (*18*), before the emergence of the Alpha, Beta, Gamma, Delta, and Omicron variants (*19*, *20*). Blood was drawn over the course of approximately 6-9 months with 5 or 6 blood draws (Fig. S1A-C and Table S1). Twenty-eight COVID-19 convalescents continued to provide blood donations after mRNA vaccination (Table S1, Fig. S1D-I). Eighteen naïve vaccinees donated up to 8 repeated blood draws after mRNA vaccination until a median of 223 days following the 2^nd^ dose (Table S1, Fig. S1D-I). Fifteen COVID-19 vaccinees and sixteen naïve vaccinees donated blood draws after their 3^rd^ mRNA dose (Table S1, Fig. S1D).

**Fig. 1.**
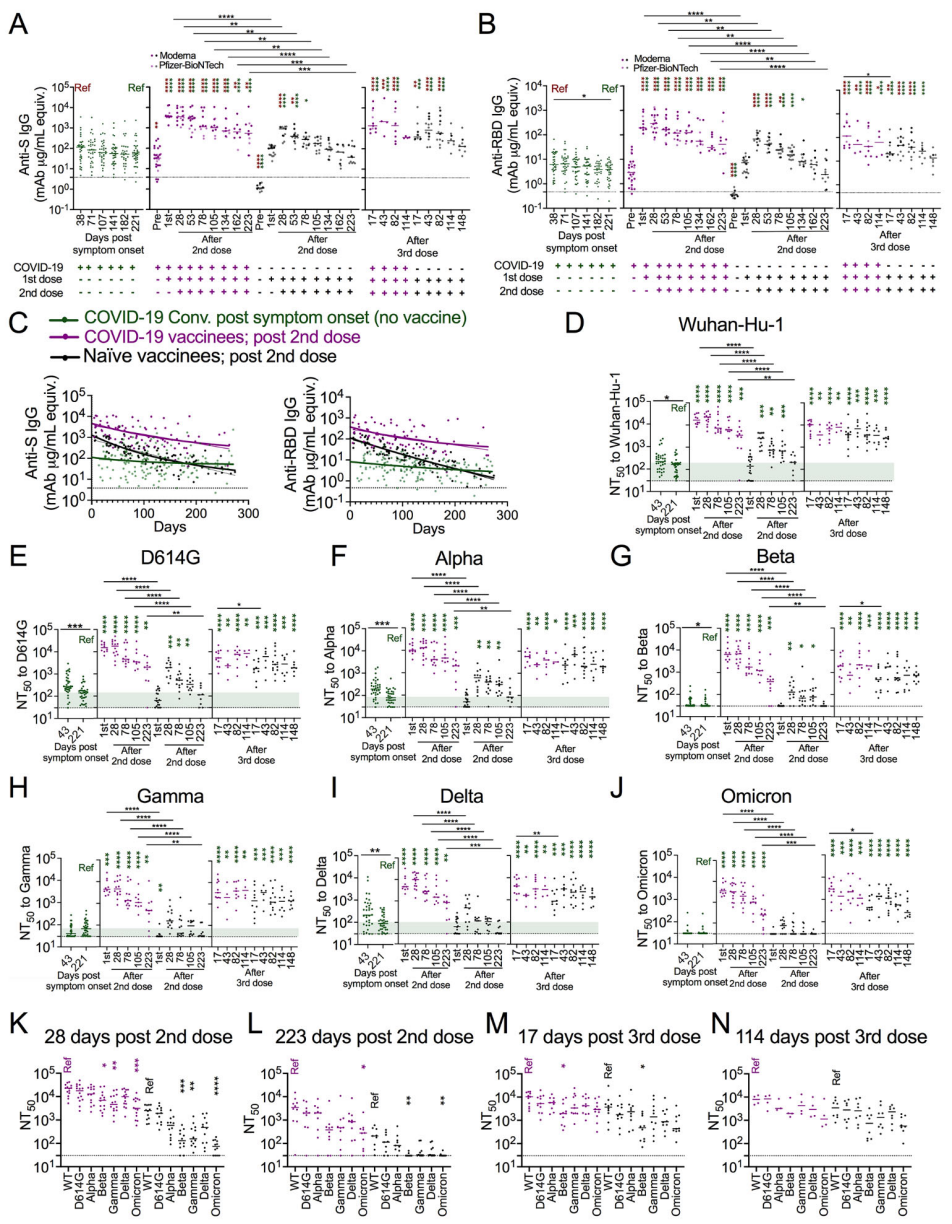
**Immune recall by vaccination after prior infection confers greater antibody magnitude and stability**. (A, B) Dot plots showing anti-spike (S) (A) and anti-RBD (B) IgG antibody levels in seroconverted COVID-19 convalescents (green, n = 28-34, left), COVID-19 vaccinees (purple, n = 4-28), and naïve vaccinees (black, n = 8-18) over time as indicated. For vaccinees, ANOVA with Tukey’s multiple comparison test was performed to test significant difference of log-transformed antibody data. (C) Dot and line graphs showing anti-S IgG (left) and anti-RBD IgG (right) trajectories in COVID-19 convalescents after natural infection (green, n = 34), and COVID-19 vaccinees (purple, n =28) as well as naïve vaccinees (black, n = 18) after the 2^nd^ vaccine dose. Overlapping fitted curves for both one phase decay (thick line) and linear regression (thin line) models are shown. Green line indicates one-phase decay model and linear regression model fitted curves for log transformed antibody data from the COVID-19 convalescents. The fitted curves from the three groups were significantly different with each other (Extra sum-of-squares F Test, p<0.0001). The Dashed lines represent twice the average of pre-COVID (negative) controls. Extra sum-of squares F test found no significant differences between one phase decay and simple linear regression fitted curves in all cases except anti-S IgG log transformed data from naïve vaccinees. Extra sum of squares F test was used to test for significance of differences between the one phase decay model fitted curves. Analysis of Covariance (ANCOVA) was performed to test the significance of differences between slopes generated from linear regression. (D-J) Dot plots showing 50% pseudovirus neutralization titers (NT_50_) to indicated SARS-CoV-2 variants in plasma collected from COVID-19 convalescents after natural infection (green, n = 34), and COVID-19 vaccinees (purple, n =3-14) as well as naïve vaccinees (black, n = 6-18) after 2^nd^ and 3^rd^ vaccination as indicated. Kruskal Wallis test and Mann-Whitney U test. (K-N) Dot plots showing NT_50_ across all the tested variants in plasma collected from COVID-19 vaccinees (purple, n =3-14) and naïve vaccinees (black, n = 8-12) at the time as indicated. Kruskal-Wallis test. Dashed lines in A-C represent twice the average of negative controls, and dashed lines in D-M represent the limit of neutralization detection (i.e., 30). Green thick ribbon indicates the median neutralization level in COVID-19 convalescents at median day of 221 days after symptom onset. Colored asterisks indicate comparisons to the corresponding colored “Ref” in each panel. *p<0.05, **p<0.01 ***p<0.001; ****p<0.0001

Anti-S and Anti-RBD IgG in COVID-19 convalescents were the most stable antibody levels over time, maintaining the majority (60-80%) of peak levels by ~220 days after symptom onset (Fig. 1A, B, Fig. S1 J, K). In addition, as expected and consistent with previous reports (*21*–*23*), one dose of mRNA vaccination boosted anti-S and anti-RBD IgG in COVID-19 vaccinees to maximal levels, beyond levels attained with two doses from naïve vaccinees (Fig. 1A, B). Both COVID-19 vaccinees and naïve vaccinees had higher peak anti-S and anti-RBD IgG than did naturally infected subjects early after SARS-CoV-2 infection (~40 days post symptom onset) (Fig. 1A, B). COVID-19 vaccinees had significantly higher anti-S and anti-RBD IgG than naïve vaccinees and naturally infected subjects out to >220 days (Fig. 1A, B). The level of naïve vaccinee anti-S IgG and anti-RBD IgG was superior to unvaccinated COVID-19 convalescent samples until ~134 days after two doses of mRNA vaccination (Fig. 1A, B).

Both linear regression and one phase decay models can fit dynamic antibody data reasonably well (*24*) and were both used here to provide insights into peak/plateau characteristics (one phase decay, thick line) and dynamic stability (linear regression, thin line) (Fig. 1C). Both models fit the data well with overlapped fitting lines (Fig. 1C). While subjects with natural infection had modest anti-S and anti-RBD IgG peak levels, over time they exhibited a high degree of dynamic stability with significantly more stable linear regression slopes than COVID-19 vaccinees and naïve vaccinees (anti-S IgG: slope = -0.001, p = 0.0047, p < 0.0001; anti-RBD IgG: slope = -0.0017, p = 0.025, p < 0.0001) (Fig. 1C, Table S2). More striking antibody decay was observed in naïve vaccinees. While anti-S and anti-RBD IgG peaked an order of magnitude higher in naive vaccinees than in naturally infected subjects, the higher levels were not maintained over time, coming down to levels on par with COVID-19 convalescent long-term plateau levels. In contrast, COVID-19 vaccinees had the highest level of peak antibodies among the three groups, as well as a more stable linear regression slope and plateau phase over time than naive vaccinees (anti-S IgG: p = 0.0268, anti-RBD IgG: p = 0.0002) (Fig. 1C, Table S2). We also analyzed plateau phase antibody dynamics by excluding the first three months (peak phase) after antigen challenge and found very similar anti-SARS-CoV-2 antibody trajectory patterns (Fig. S2A, Table S2). In general, the Pfizer–BioNTech vaccine induced slightly lower anti-S and anti-RBD IgG than the Moderna vaccine, but the difference did not reach statistical significance in these cohorts (Fig. S2B and C) and the trajectory dynamics were similar (Fig. S2D and E). A homologous 3^rd^ dose of mRNA vaccination in COVID-19 naïve individuals boosted antibody levels and cross-variant neutralization function that remained robust throughout median 148 days post 3^rd^ dose (Fig. 1A, B, D-J).

To examine functional antibody breadth, we compared cross-variant neutralization in the plasma using pseudotyped virus neutralization assays we have previously shown to mirror that seen with authentic virus (*25*). We found that COVID-19 vaccinee plasma had greater cross-variant neutralization function than did plasma from naïve vaccinees and unvaccinated COVID-19 convalescents at all tested time points (Fig. 1D-J, Fig. S3 A-G), consistent with greater anti-S and anti-RBD IgG levels in this group. By 223 (median) days after the 2^nd^ dose, neutralization levels to all variants in naïve vaccinees decreased to the level that naturally infected subjects had at ~220 days after symptom onset (Fig. 1D-J). After the 3^rd^ dose of mRNA vaccination, the cross-variant neutralization level, including to Omicron, was greatly enhanced in naïve vaccinees (Fig. 1D-N). These data suggest that pre-existing immunity from natural infection primed greater vaccine-induced magnitude as well as less long-term antibody decay.

### Greater cross-variant neutralization equality induced by natural infection and vaccination

**Fig. 2.**
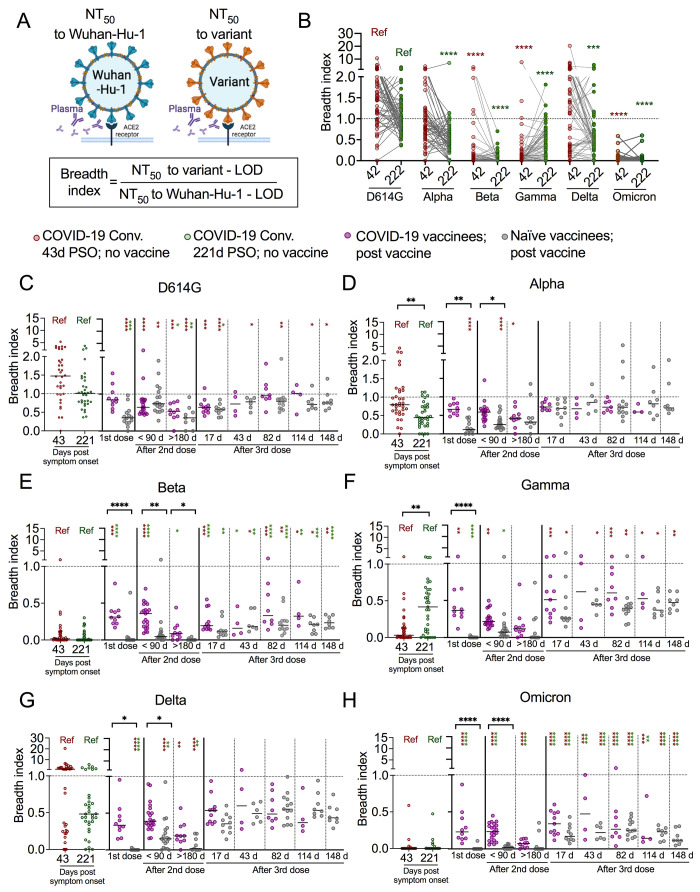
Greater cross-variant neutralization equality induced by natural infection and a 3^rd^ mRNA vaccine dose. (A) Schematics to show the breadth index calculation, which is the ratio of the 50% neutralization titer (NT_50_) to SARS-CoV-2 variant subtracted limit of detection (LOD) divided by NT_50_ to the original Wuhan-Hu-1 strain subtracted LOD of the same plasma sample. (B) Dot and line graphs of calculated breadth indexes to the indicated variants in plasma polyclonal neutralizing antibodies collected at median 42 days (red, n = 59) and 222 days (green, n = 57) after COVID-19 symptom onset in COVID-19 convalescents without vaccination follow up (cohort A) and COVID-19 convalescents before vaccination (cohort B, pre-vaccination). Kruskal-Wallis test. (C-H) Dot plots showing breadth indexes to the indicated SARS-CoV-2 variants in plasma collected from COVID-19 convalescents without vaccination follow up (n = 33-34), COVID-19 vaccinees (purple, n = 3-21), and naïve vaccinees (grey, n = 6-18) at the median time points (days) post symptom onset or vaccination as indicated. Red and green asterisks indicate comparisons to early (red) and late (green) COVID-19 convalescent breadth indexes as indicated by the red and green “Ref”. Kruskal-Wallis test was performed to test significance in multiple comparisons. *p<0.05, **p<0.01, ***p<0.001; ****p<0.0001.

The cross-variant evenness of neutralization potency appears to vary between early and late time points (Fig. 1 K and L) and neutralization function appears to be strikingly more equal across all tested variants shortly after a 3^rd^ mRNA dose (Fig. 1 M and N). To quantify the evenness of cross-variant neutralization function of antibody repertoires over time, we calculated a breadth index, which is the quotient of 50% neutralization titer (NT_50_) to each variant, divided by NT_50_ to original Wuhan-Hu-1 strain for each plasma sample (Fig. 2A). The assay limit of detection (NT_50_ = 30) was subtracted from each NT_50_ value before division for background correction. Breadth index values were plotted for each variant where Wuhan-Hu-1 was above the limit of detection (94.3% of samples) (Fig. 2B-H). The minority of samples (5.7%) where NT_50_ to Wuhan-Hu-1 was at the limit of detection (i.e., breadth index denominator zero) were also at or near the limit of detection for all variants and are shown separately (Table S3). We also confirmed that background-corrected breadth index calculations were not influenced by neutralization potency (Fig. S3H).

From ~40 days to ~220 days post symptom onset, the breadth index in plasma collected from subjects after natural infection evolved over time (Fig. 2B-H). Compared to samples collected early after infection, COVID-19 convalescent samples had significantly greater neutralization breadth to Gamma, and lower neutralization breadth to Alpha ~220 days post symptom onset (Fig. 2B-H). Neutralization function against the Omicron variant was lowest among all the tested variants (Fig. 2B).

When comparing COVID-19 convalescent individuals and COVID-19-naïve participants after vaccination, we found unvaccinated COVID-19 convalescents had significantly greater breadth indexes to Gamma and Delta than naïve vaccinees (Fig. 2C-H). In addition, vaccination of COVID-19 convalescents provided further enhancement of the breadth index for Beta and Omicron (Fig. 2C-H, Fig. S3I-N) early after the 2^nd^ dose. The differences of breadth indexes to variants between COVID-19 vaccinees and naïve vaccinees were most striking early after the 2^nd^ dose, and gradually decreased afterwards (Fig. 2C-H). The 3^rd^ dose in naïve vaccinees significantly increased the breadth indexes to all the VOCs (Fig. 2C-H). These results indicate that natural infection elicited a polyclonal antibody response with greater recognition breadth than two doses of mRNA vaccination in naïve vaccinees. The greater infection-induced cross-variant neutralization evenness was maintained or enhanced after vaccination. Notably, the 3^rd^ mRNA dose in COVID-19-naïve vaccinees enhanced cross-variant neutralization evenness to levels with stability that approximated the hybrid immunity conferred by infection plus vaccination (Fig. 2C-H).

### Early memory B cell cross-coronavirus reactivity is a signature of greater antibody durability

Because prior SARS-CoV-2 infection enhanced durability after vaccination, we considered whether prior endemic coronavirus infection may have influenced differential antibody durability in our convalescent cohort. We previously reported two general subsets of COVID-19 convalescents with respect to antibody dynamics over time, antibody sustainers and antibody decayers (*18*). Sustainers exhibited the same or increasing antibody levels over time while decayers were defined as those that lost antibody levels over the same time frame. We used earliest available blood draws (~40 days post symptom onset) to ask whether there are any differences in cross reactivity to other coronaviruses in the early post-infection memory B cell compartment of sustainers versus decayers.

**Fig. 3.**
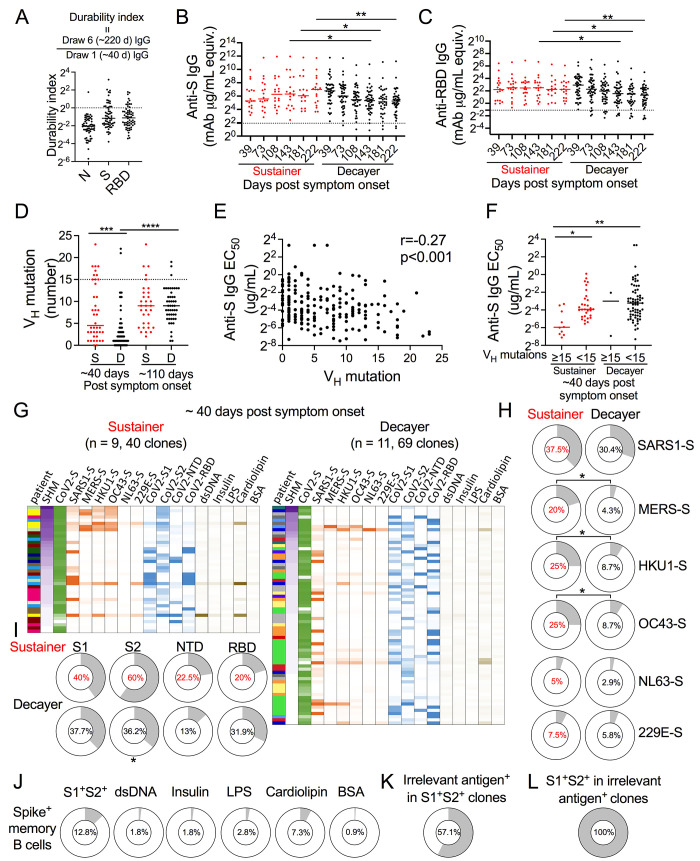
Early memory B cell cross-coronavirus reactivity is a signature of greater antibody durability. (A) Dot plot illustrating anti-nucleocapsid (N), spike (S) and RBD antibody durability indexes from seroconverted subjects (n=62). The durability index was reported as the ratio of the last draw (6^th^ draw) IgG level to the first draw IgG level of same subject. Dashed line represents durability index of 1. (B, C) Dot plots showing anti-S (B) and anti-RBD (C) IgG levels in sustainers (anti-S IgG durability index ≥ 1, red, n = 16-18) and decayers (anti-S IgG durability index <1, red, black, n = 37-44). Dashed lines represent twice the average of pre-COVID controls. Mann-Whitney U test. Unpaired *t* test after log-transformation of antibody data. (D) Dot plot showing mAb heavy chain V gene segment (V_H_) mutation number per sequence cloned from S^+^ memory B cells in sustainer (S, red) and decayer (D, black) collected ~ 40 days after symptom onset (sustainer, n = 9, 40 clones; decayer, n = 11, 69 clones;1^st^ draw) and ~110 days after symptom onset (sustainer, n = 8, 32 clones; decayer, n = 9, 48 clones; 3^rd^ draw). Kruskal-Wallis test. (E) Scatter plots illustrating Spearman correlation between anti-S IgG EC_50_ and V_H_ mutation in all mAbs described in (D). (F) Half-maximal effective concentration of binding (EC_50_) data (see methods) of sustainer-isolated (red) versus decayer-isolated (black) anti-S mAbs with greater than or less than 15 V_H_ mutations as indicated. The mAbs were isolated ~ 40 days after symptom onset. Kruskal-Wallis test. (G) Heatmap of SHM and binding analysis of mAbs cloned from sustainers (left) and decayers (right) collected ~40 days after symptom onset as described in (D). mAbs are listed in order of highest to lowest SHM. SHM amount is indicated by color (purple) intensity. Different colors in the first column for each panel indicate different individuals. Green color intensity indicates mAb binding avidity to SARS-CoV-2 spike from flow cytometry-based EC_50_ measurement. Orange indicates mAb binding to human coronavirus spikes. Blue indicates mAb binding to SARS-CoV-2 subunits. Brown indicates mAb binding to irrelevant antigens. Intensity of color indicates OD_405_ values. (H-J) Donut charts showing percentages of mAb binding to the indicated HCoV spikes (H) and SARS-CoV-2 S subdomains (I) in sustainer (red) and decayer (black) groups as described in (G). Fisher exact test. (J) Donut plots showing percentages of S1 and S2 dual mAb binders and the mAb binding to the indicated irrelevant antigens in SARS-CoV-2 S^+^ memory B cells isolated ~40 days post symptom onset in COVID-19 convalescents as described in (G). (K) Donut plots showing percentages of mAbs binding to any of the irrelevant antigens (dsDNA, Insulin, LPS, Cardiolipin, and BSA) as shown in (G) in S1^+^S2^+^ dual binders (14 clones). (L) S1^+^S2^+^ dual binders in mAbs binding to any of the irrelevant antigens (8 clones). *p<0.05, **p<0.01, ***p<0.001.

As described previously (*18*), we generated durability indexes by taking virus-specific IgG magnitude values from the most recent blood draw (6^th^, median 222, range 185-271 days post symptom onset) divided by the respective IgG levels of the 1^st^ draw (median 39, range 13-85 days post symptom onset) for nucleocapsid (N), S and RBD (Fig. 3A). Anti-S IgG durability index grouped COVID-19 convalescents into long-term sustainer (durability index ≥1, n = 18) and decayer (durability index < 1, n = 44). Sustainers had shorter symptom duration, milder disease severity and tended to be younger as we reported previously (Fig. S4A) (*18*). The sustainers had more anti-S and anti-RBD IgG than decayers, for up to 9-months after symptom onset (Fig. 3B and C).

T cell cross reactivity analysis revealed that, while sustainers demonstrated higher frequency of non-naïve CD4^+^ (CD45RA^-^CD4^+^) T cells than decayers (*18*), there were no significant differences in antigen specific CD4^+^ T cell or CD8^+^ T cells between sustainers and decayers (Fig. S4B-G) as determined by activation induced marker (AIM) assays (*26*, *27*). A more sensitive tetramer-based approach found increased S^+^ memory circulating CD4^+^ T follicular helper cell (cTfh) frequencies in anti-S antibody sustainers (*28*), suggesting a connection between S-specific CD4+ T cell responses and anti-S antibody durability.

To assess antibody cross-reactivity to other coronaviruses, we isolated S^+^ memory B cells (Fig. S5A), cloned antibody genes, and purified their monoclonal antibodies (mAb) from sustainers and decayers ~40 and ~110 days post symptom onset (*18*). We previously showed that by ~110 days, sustainers had higher SHM in their S^+^ memory B cell antibody genes early after infection (~40 days) than decayers (*18*). New analysis revealed that these sustainers retained anti-S or anti-RBD durability indexes at levels ≥1 to at least ~220 days after symptom onset (Fig. 3A). We produced mAbs from which we were able to recover paired heavy and light chain sequences from single cell antibody cloning (Table S4). While there was no overall difference in V_H_ gene segment usage, sustainers showed a higher level of SHM at day ~40 (Fig. 3D, S5B and C), like the sequence analysis of the overall pool (i.e., including non-paired IgH and IgL sequences) (*18*). When compared to mAbs cloned and produced from samples drawn at ~40 days post symptom onset, those from ~110-day samples had higher SHM as expected (*18*).

We examined the binding of each mAb to spike expressed on 293T cells by flow cytometry and estimated their avidity by calculating half-maximum effective concentration of binding (EC_50_) (Fig. S5D and E). We found that S^+^ mAb EC_50_ were significantly negatively correlated with IgH V gene mutations, indicating that more highly mutated mAbs had higher avidity for S (Fig. 3E). Sustainers had significantly greater number of highly mutated clones with at least 15 IgH mutations ~40 days after symptom onset, as expected (*18*) (Fig. 3F).

mAb binding analysis showed that sustainers harbored significantly higher frequencies of memory B cells reactive with endemic human coronavirus (HCoVs) HKU1 and OC43 spikes, which tended to have higher SHM (≥15) with high avidity to S (Fig. 3F), and map to S2 (Fig. 3G and H). We selected SHM≥15 since it appeared to be the neck of a bi-modal distribution for heavy chain SHM in sustainers early after infection. In addition, sustainers had significantly higher frequency of memory B cells binding to S2 (Fig. 3G, I), which is highly conserved across coronaviruses (*29*–*31*). We found 12.8% of the S^+^ memory B cells react to both S1 and S2, which we interpret to be due to polyreactivity. This is consistent with prior work approximately 1 out of 5 IgG^+^ memory B cells in the human repertoire is polyreactive (*32*). Consistent with this interpretation, we find that over half (57.1%) of S1 and S2 dual binding mAbs bind to an irrelevant antigen and all the S-binding mAbs that bind an irrelevant antigen bind both S1 and S2 (Fig. 3J-L). No significant differences were observed in the amounts of plasma antibody to coronavirus spikes between sustainers and decayers (Fig. S5F).

**Fig. 4.**
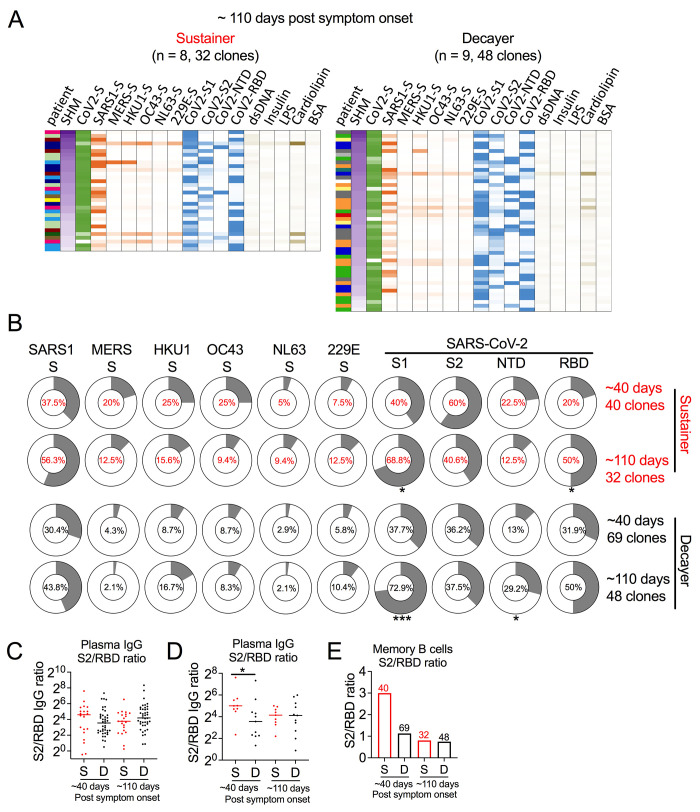
Evolution of antibody recognition to coronaviruses in COVID-19 convalescents. (A) Heatmap of SHM and binding analysis of mAbs cloned from sustainers (left) and decayers (right) collected ~110 days after symptom onset as described in Fig. 3G. (B) Donut plots showing the binding frequency of sustainer-isolated (red) versus decayer-isolated (black) S^+^ memory B cell-derived mAbs to the indicated coronavirus spike proteins. Memory B cells were collected ~ 40 days after symptom onset (sustainer, n = 9, 40 clones; decayer, n = 11, 69 clones;1^st^ draw) and ~110 days after symptom onset (sustainer, n = 8, 32 clones; decayer, n = 9, 48 clones; 3^rd^ draw). Fisher exact test. (C) Dot plot showing the ratio between plasma anti-S2 IgG and anti-RBD IgG from all anti-S IgG or anti-RBD IgG sustainers (n = 21) and decayers (n = 41) at ~40 or ~220 days after symptom onset. Mann-Whitney U test. (D) Dot plot showing the ratio between plasma anti-S2 IgG and anti-RBD IgG from sustainers (n = 9) and decayers (n = 11) selected for memory B cell sorting at ~40 or ~220 days after symptom onset. Selection was based on similar initial antibody levels. Mann-Whitney U test. (E) Bar graph showing the ratio of S2-binding mAb frequencies to RBD-binding mAb frequencies in sorted memory B cells from sustainers (S, red) and decayers (D, black) at ~40 or ~220 days after symptom onset. Number of mAbs in this analysis are indicated on the Bar graph. *p<0.05, ***p<0.001.

At ~110 days after symptom onset, the frequency of S2 and endemic coronavirus cross-reactive clones tended to decrease; and the frequency of S1 and RBD binding clones, increase (Fig. 4A and B). The S2/RBD plasma IgG ratio in sustainers early after infection tended to be greater in sustainers than decayers at ~40 days in the total population (Fig. 4C)—a trend that achieved significance in plasma linked to the subset of individuals from which antibodies of S^+^ memory B cells were cloned and expressed (Fig. 4D). The greater S2/RBD IgG in early, but not late sustainer plasma after infection was mirrored in a similar S2/RBD ratio analysis of sustainer and decayer memory B cells (Fig. 4E). These findings are consistent with the notion that higher relative proportion of anti-S2 IgG in sustainers early after infection may suppress recalled S2-binding cross-reactive memory B cell frequencies over time due to an epitope masking phenomenon by pre-existing anti-S2 antibodies, driving a change in immunodominance over time (*33*). Whereas the S2-binding mAbs didn’t show difference in V_H_ gene segment usage compared to other subdomain binding mAbs (Fig. S6A and B), these mAbs harbored higher percentage of clones sharing the same heavy and light chain V gene segments (Fig. S6C). In addition, we found examples of clonal expansion (mAbs with same V_H_, V_L_ and CDR3s from the same donor) and convergent clones (mAbs with same V_H_, V_L_ and >50% CDR3 similarity from different donors) in the cross-reactive mAb pool (Fig. S6D). Together, these data suggest that early cross-reactive memory B cells recalled from prior endemic coronavirus infection is linked to stronger antibody durability over time.

### Anti-S IgG sustainers produced superior cross-variant neutralizing capability

**Fig. 5.**
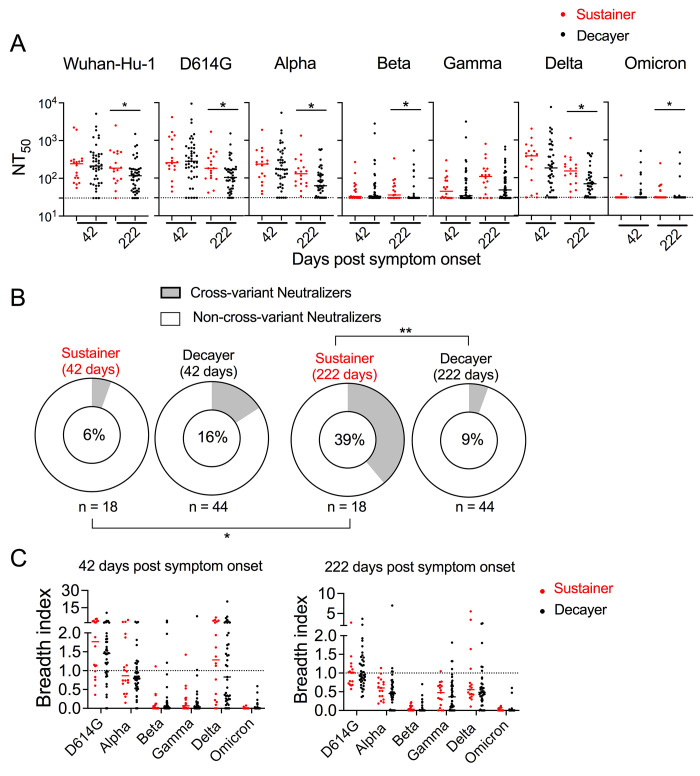
Anti-S IgG sustainers produced superior cross-variant neutralizing capability over time. (A) Dot plots showing pseudovirus neutralization titers to the indicated SARS-CoV-2 variants in plasma collected at the indicated times (median 42, range 13-119; median 222, range 185-271) from sustainers (n = 18, red) and decayers (n = 44, black). Mann-Whitney U test. (B) Donut plots illustrating percentages of cross-variant neutralizers at the indicated times after symptom onset. Subjects were scored as cross-variant neutralizers if their plasma neutralization titer was more than the detection limit (i.e., 30) for all the variants tested in (A). Fisher exact test. (C) Dot plots showing breadth indexes of plasma collected from anti-S IgG sustainer (red) and decayer (black) at the indicated time after symptom onset. Mixed effect analysis. *p< 0.05, **p< 0.01.

We evaluated sustainer versus decayer cross-variant SARS-CoV-2 neutralization ability of the plasma collected ~40 days (median 42, range 13-119) and ~220 days (median 222, range 185-271) after symptom onset (Fig. 5A and S7A). While there was no significant NT_50_ difference in plasma collected ~40 days after symptom onset between sustainers and decayers, neutralizing antibody levels to original Wuhan-Hu-1 strain as well as variants of concern Alpha, Beta, Delta, and Omicron were significantly greater in sustainers compared to decayers long after (~220 days) infection as expected (Fig. 5A).

When tested early after COVID-19 recovery (~40 days post symptom onset), 6% of the sustainer and 16% of decayer plasma samples exhibited cross-neutralization function to all the tested strains (Fig. 5B). In contrast, 39% of sustainers and 9% of decayers had cross-variant-neutralizing function to all tested strains ~220 days after symptoms onset (Fig. 5B). More detailed neutralization analysis suggested decayers had more severe loss of neutralization capacities against the Alpha, Beta, Delta, and Omicron variants ~220 days after symptom onset (Fig. S7A). As expected, correlation analysis indicated that cross-variant neutralization superiority in sustainers is linked to anti-S and anti-RBD antibody levels (Fig. S5B and C) highlighting the value of durable antibody levels over time. Vaccination after infection in sustainers and decayers normalized the antibody and cross-variant neutralization differences in these two groups (Fig. S8).

There were no differences in neutralization breadth indexes between sustainers and decayers at ~40 or ~220 days post symptom onset (Fig. 5C), suggesting that the greater absolute neutralization breadth observed in sustainers at later time points is more likely due to greater antibody magnitudes of similar cross-variant repertoire recognition composition. These results underscore an important role for anti-S antibody dynamic stability in the durable resistance to emerging SARS-CoV-2 variants.

## DISCUSSION

Inducing long lasting immune memory through widespread vaccination is critical to maximize protection from COVID and other infectious threats. Obstacles to robust immune memory include waning antibody levels and immune escape through viral evolution. It is therefore critical to better understand how to elicit potent, long-lasting immunity broadly effective across evolving viral variants through vaccination. Data here suggest that immune recall through both vaccination and infection confers greater antibody durability and breadth to SARS-CoV-2 variants. In addition, early memory B cell cross-reactivity is identified as a marker for more sustained antibody responses after infection.

Compared to a two-dose mRNA vaccine, a history of SARS-CoV-2 infection provided more even spike-specific recognition across SARS-CoV-2 variants that had not yet emerged at the time of infection. The greater evenness of anti-spike neutralization function was carried forward in antibody responses in COVID-19 convalescents after vaccination. Strikingly, a homologous 3^rd^ injection induced much more even cross-variant recognition than two doses in naïve vaccinees, including to Omicron as also seen by other recent studies (*34*–*36*), which we show here to be on par with that of COVID-19 convalescents and COVID-19 vaccinees.

In addition to more even cross-variant recognition, more gradual decay was also seen in convalescent individuals following vaccination. Within the COVID-19 convalescent cohort, improved antibody durability surfaced in those with cross-coronavirus reactive anti-S2 IgG in the memory B cell compartment as well as relatively greater early anti-S2 secreted antibody responses. This suggests that B cell memory to endemic coronaviruses may be a prelude to enhanced stability of anti-SARS-CoV-2 antibody responses.

Because long-term antibody secretion is supported by LLPCs, these data may provide insights into optimal triggers for LLPC development. LLPCs are the sources of long-lived secreted antibodies responsible for months/years-long stable antibody plateau levels and tend to have relatively high levels of SHM (*37*–*39*). The magnitude of the stable virus-specific IgG plateau phase after infection or vaccination is likely proportional to the number of sourcing LLPCs induced at the time of immune challenge. In this light, a deeper understanding the ground rules underlying LLPC fate commitment will be important to guide vaccine strategies to maximize durability.

We previously described convalescent individuals who sustained antibody responses healed quicker and had greater early memory B cell somatic mutation levels (*18*). Here we report that sustainers also had significantly greater early cross-coronavirus reactivity in their memory B cell pool and that nearly all the cross-coronavirus reactivity was in the highly mutated (SHM ≥ 15) memory B cells. This shows a clear link between endemic coronavirus cross-reactivity and long-term antibody durability induced by SARS-CoV-2.

One interpretation is that, in addition to leaving an imprint in the memory B cell pool, prior and/or recent endemic coronavirus infection may have resulted in an immune system better prepared to handle SARS-CoV-2 invasion (shorter symptom duration) and support an environment more conducive for LLPC differentiation (durable antibody response). Recent findings showing greater spike specific cTfh cells enriched in sustainers (*28*) is consistent with the possibility that sustainers may be equipped for a more robust GC response and memory retention. Noteworthy is that essentially all the highly mutated, cross-reactive memory B cells bind S2 and not RBD. So, the fact that anti-RBD antibodies also showed a significantly greater degree of durability in sustainers suggests that LLPC differentiation preference likely involved more than just a simple model of greater LLPC differentiation from recalled memory B cells, since RBDs are very poorly conserved across different coronaviruses.

Prior exposure to endemic human coronavirus is very common (*40*), but only a subset of individuals harbored substantial cross-reactive memory B cells. It’s not clear whether this reflects recent infection or whether sustainers are intrinsically different in cross-reactive memory B cell retention. A limitation of this study is the lack of pre-COVID-19 samples from sustainers and decayers.

Achieving and maintaining robust antibody production can have a positive effect on recognition breadth across a diversity of SARS-CoV-2 variants. Antibody magnitude correlates with variant recognition breadth likely because human pre-VOC infection-induced S-specific memory B cell repertoires includes a low but reliable frequency of specificities that neutralize broadly across variants of concern (*25*). In addition to altering cross-variant breadth by differing magnitudes of the same general antibody repertoire composition, the frequency of variant S-reactive antibodies within a polyclonal composition may also differ between groups. Our measurement of an antibody breadth index normalizes for antibody neutralization potency—providing a measure of evenness of functional cross-variant recognition per unit potency. The heterogeneity of breadth index calculations between different antigen exposure histories suggests that vaccines and/or vaccine strategies may be strategically designed to enrich antibody responses that maximize anti-variant neutralization breadth.

The immunological scenario in connection to endemic coronavirus memory B cell recall and antibody durability/breadth after infection may be different from vaccination. In addition, although both infection and vaccination cohorts shared similar pre-VOC SARS-CoV-2 S glycoproteins as antibody targets, polyclonal anti-SARS-CoV-2 variant neutralizing antibody recognition breadth indexes evolved differently over time after natural infection compared to mRNA vaccination. It is possible that replicating intact viruses in natural infection may elicit more even cross-variant anti-spike antibody recognition coverage than the coverage elicited by the stabilized version of spike in current vaccines. However, because a homologous 3^rd^ dose of mRNA encoding spike induces much more even cross-variant recognition than post 2^nd^ dose, recalled immunity delivers a different memory composition, likely influenced by antibody affinity maturation that continues to occur in the months-long interval between 2^nd^ and 3^rd^ vaccine doses (*41*).

In this context, immune responses early after SARS-CoV-2 infection and vaccination have been characterized by an initially low-SHM spike-reactive memory B cell pool that slowly evolves over months to higher SHM and greater antibody affinity (*18*, *42*–*44*). Since the initial human memory B cell pool harbors consistent, but low-potency neutralizing antibodies to more conserved spike targets (*25*), and because antibody affinity correlates with neutralization potency (*45*), the state of memory B cell maturation at the time of recall is a likely contributing determinant of the degree of cross-variant neutralization breadth that can emerge after homologous boosting. More mature B cell memory may also influence longevity as memory B cells with higher affinity toward immunizing antigen are preferred candidates to differentiate into antibody secreting cells in response to a subsequent boosting dose (*46*). Thus, a greater frequency of more mature, high affinity memory B cells may also contribute to increased probabilities of longer-lived plasma cell production.

## MATERIAL AND METHODS

### Study design

This study was designed with the goal to assess the impact of recalled immunity in human on anti-SARS-CoV-2 antibody durability and recognition breadth to variants. We characterized the plasma anti-S IgG and anti-RBD IgG antibody level as well as their neutralization level toward original Wuhan-Hu-1 stain and variants (D614G, Alpha, Beta, Gamma, Delta, and Omicron) in three longitudinal cohorts being followed for more than 200 days: (i) pre-VOC infection alone individuals; (ii) pre-VOC infection plus mRNA vaccination; and (iii) COVID-19 naïve individuals with mRNA vaccination. Following the individuals with infection and/or mRNA vaccination, we modeled the anti-S and anti-IgG antibody trajectory to assess the antibody durability, and generated breadth indexes to evaluate the evolving of neutralization evenness across the variants. To assess whether prior endemic coronavirus infection may influence differential antibody durability in infection alone subjects, we compared endemic coronavirus antibody level, T cell cross-reactivity and memory B cell cross-reactivity in individuals harboring sustained or decayed anti-SARS-CoV-2 antibody. After selection of sustainers and decayers based on their similar initial antibody level, we performed T cell and memory B cell cross-reactivity analysis. We produced spike-binding mAbs from memory B cells of these individuals and compared SHM, EC_50_, V_H_ gene segment usage, clonal relationships, and their reactivity to a panel of human coronavirus spike and autoantigens. Finally, we evaluated the cross-variant neutralization function in the plasma isolated from these subjects.

### Human subjects

Mass General Brigham Institutional Review Board approved this study and protocol. We recruited participants in Boston area through online advertisement on institutional clinical study website open to public and flyers posted in local hospitals (*18*). Adult volunteers with a history of COVID-19 were recruited between March 2020 and June 2020. As described before (*18*), the eligibility criteria include symptom diagnosis by health professionals and a SARS-CoV-2 positive nasopharyngeal swab RT-PCR test. After enrollment, clinical team identified one subject was not diagnosed by nasopharyngeal swab RT-PCR test, but by Quest Diagnostics antibody test and symptoms consistent with COVID-19 (*18*). Participants self-rated their severity of COVID-19 symptoms with a scale from 1 (very mild) to 10 (very severe). They self-reported their symptom onset date and recovery date, with symptom duration being the difference between the two dates. Sixty-two subjects donated 5 or 6 repeated blood draws until middle of December 2020 before they start getting vaccinated. Demographic information is provided in Table S1. Twenty-eight COVID-19 convalescents continued donating blood after they received BNT162b2 or mRNA-1273 vaccines. Demographic information for these individuals is provided in Table S1.

The subjects participated in the vaccination study were recruited in Boston Area after vaccine emergency use authorizations as described above. Blood draws from participants before vaccination were collected when possible. Participants self-reported vaccination type, vaccination dates, any history of COVID-19 diagnosis and other diseases including immunocompromised status due to medical condition or medication, lung disease, diabetes, and cardiovascular disease. Vaccinated subjects who reported no history of a COVID-19 diagnosis were designated as naïve vaccinees, and this was confirmed with anti-N IgG screening. One participant screened positive for anti-N IgG but was negative for anti-S IgG and anti-RBD IgG within 7 days of the first dose, so was considered a naïve vaccinee. Blood draws collected before or within one day of the 1^st^ dose were designated as pre-vaccination draws. A blood draw for each subject collected after the 1^st^ vaccine dose and before or within 4 days of the 2^nd^ vaccine dose were designated as post 1^st^ dose blood draws. Detailed demographic information describing the naïve vaccinees in this study are provided in Table S1.

### Plasma and PBMC isolation from blood

Blood from participants were collected in EDTA tubes, which were centrifuged at 1200 rpm for 10 min to collect the plasma in the upper layer. Plasma was further spun for 10 min at 2200 rpm to remove debris, and then divided into aliquots to store at -80°C. The lower layer was diluted 1:1 in PBS and loaded on Ficoll (GE lifescience) for PBMCs separation at 2200 rpm for 20 min. PMBCs were washed once in PBS and then resuspended in FBS containing 10% DMSO and aliquoted for -80°C and liquid nitrogen storage.

### Cell lines

HEK293T cell line (ATCC) was maintained in DMEM containing 10% FBS, 100 U/mL penicillin and 100 U/mL streptomycin (Gibco). HEK293T stable cell line overexpressing human ACE2 and TMRPSS2 (ACE2/TMPRSS2-expressing HEK293T) was kindly provided by Dr. Marc Johnson (University of Missouri School of Medicine). All cell lines were incubated at 37°C with 5% CO_2_.

### Recombinant coronavirus proteins

SARS-CoV-2 RBD was kindly provided by Aaron Schmidt’s lab (Ragon Institute of MGH, MIT and Harvard). SARS-CoV-2 Nucleocapsid was purchased from Genscript (Z03480). SARS-CoV-1 spike (40634-V08B), MERS spike (40069-V08B), HKU1 spike (40606-V08B), OC43 spike (40607-V08B), NL63 spike (40604-V08B), 229E spike (40605-V08B), SARS-CoV-2 NTD (40591-V49H) and S2 (40590-V08B) proteins were purchased from Sino Biological.

### Generation of plasmids expressing SARS-CoV-2 variants

We designed 5′ and 3′ primers specific for each variant spike (Alpha, Beta, Gamma, Delta, each supplied by the Bing Chen laboratory), and amplified the variant sequences by Phusion® High-Fidelity DNA Polymerase (Thermo fisher). We amplified Alpha, Gamma and Delta variant spike with forward primer 5′-TGGCAAAGAATTCCGCCACCATGTTCGTGTTTCTGGTGCTGCTG-3′ and reverse primer 5′-GGCCCTCGAGGTCGACGGTATCGATAAGCTTTCATTAGCTGCCACAGCTACAACAGC-3′. We amplified Beta variant spike with forward primer 5′-TGGCAAAGAATTCCGCCACCATGTTCGTGTTTCTGGTGCTGCTG-3′ and reverse primer 5′- GGCCCTCGAGGTCGACGGTATCGATAAGCTTTCATTAGGAGCCACAGCTGCAACAGC -3′. The omicron spike gene was synthesized at IDT. We cloned each variant into pHDM vector through EcoRI and SalI restriction enzyme cutting site. To be consistent with the pHDM-Wuhan-Hu-1-delta21 (155130) and pHDM-D614G-delta21 (158762) plasmids obtained from Addgene, the c-terminal 21 amino acid on all the variants were deleted (*47*).

### Flow cytometry and single cell sort

Frozen PBMCs were quickly thawed at 37°C and transferred to warm RPMI containing 10% FBS. S^+^ memory B cells were single sorted (BD FACSAria Fusion) into 96 well PCR plates as described before (*18*). Briefly, B cells were enriched by anti-CD19 magnetic beads (Miltenyi) and flow through was collected for T cell analysis. Enriched B cells were stained with Flag tagged SARS-CoV-2 spike (Genscript, Z03481) then incubated with APC conjugated anti-Flag and PE conjugated anti-Flag for double staining. DAPI^-^IgM^-^IgD^-^IgG^+^CD27^+^Spike^+^ cells were single sorted into 96 well PCR plates with 4μL/well lysis buffer (0.5x PBS containing 10mM DTT, and 4 U RNaseout) and stored at -80°C for further analysis (*48*).

### Antibody sequencing, cloning and expression

RNA from single-sorted S^+^ memory B cell were reverse transcribed into cDNA followed by IgH, Igκ, and Igλ gene amplification through semi-nested PCR as described (*18*, *48*). The PCR products from second amplification were sent for Sanger sequencing. Sequences were filtered through GENEWIZ-provided quality score, then aligned by IgBlastn v.1.16.0. V gene segment-specific forward primers and J gene segment-specific reverse primers were used in the PCR reactions to amplify specific antibody genes from the 1^st^ round PCR products. PCR products were cut with restriction enzymes, purified, then ligated into expression vectors (*48*). Ligation products were then transformed in *E.coli* Top10 competent cells (Thermo fisher) to generate colonies which were picked for plasmid extraction and sanger sequencing. Once plasmid sequences were confirmed through Igblast and aligned with the original 2^nd^ round PCR product sequences, paired IgH and IgL plasmids were transfected into 293T cells by lipofetamine 3000 (Thermo fisher). Supernatants from 293T cells were harvested 6 -7 days after transfection and screened in the ELISA for IgG expression. Supernatants with IgG expression were purified by protein A (Thermo fisher), eluted in 150 μL 0.1 M glycine (pH 2.8), and collected in 15 μL 1 M Tris (pH 8.0) (*48*, *49*). Elution was further dialyzed in sterile PBS every three hours for three times based on manufacturer’s instructions (Thermo fisher, 88260). Concentrations of purified antibody were measured by Nanodrop (Thermo fisher), and stored at 4°C.

### ELISA

Plasma IgG quantification of coronavirus antigens were performed as previously described (*18*). Briefly, 96 well maxisorp ELISA plates (Thermo fisher) were coated with 2 μg N protein, 2 μg S protein, or 2 μg RBD protein in 30μL PBS overnight at 4°C. After discarding coated solutions, ELISA plates were blocked in 100 μL 3% BSA for 2 hours. During the incubation time, plasma was transferred to tubes and combined with equal volume of 2% Triton in PBS and incubated 20 min at room temperature for inactivation. Plasma from COVID-19 convalescents were serially diluted 2 times from 1:100 to 1:6400, and post-vaccination plasma samples were serial diluted 3 times from 1:100 to 1:72900 in PBS containing 1% BSA and 0.05% Tween20. For the anti-S and anti-RBD antibody standard, the monoclonal antibody CR3022 was serially diluted 2 times from 0.5 μg/mL. After blocking solution was discarded, ELISA plates were washed once in PBS containing 0.05% Tween20. The plasma dilutions were transferred to ELISA plates with duplicates and standards included on each plate as controls. After overnight incubation at 4°C, ELISA plates were washed three times in PBS containing 0.05% Tween20, and then incubated 90 min with alkaline phosphatase (AP) conjugated anti-human IgG (Southern biotech) diluted at 1:1000 in PBS containing 1% BSA and 0.05% Tween20. ELISA plates were washed three times in PBS containing 0.05% Tween20, and then incubated with 100 μL alkaline phosphatase substrate solution (Sigma) for 2 hours. Plates were read at 405 nm through a microplate reader (Biotek Synergy H1). Graphpad software generated standard curves for each plate by non-linear regression. The plasma Ig level was interpolated from a single dilution with OD_405_ values falling in the middle range of the standard curve. Antibody binding values were reported as CR3022 monoclonal antibody concentration equivalents (mAb μg/mL equivalent). Four pre-COVID controls were included on each plate. Samples were scored positive if the antibody level was higher than 2x average of the pre-COVID controls for each antigen. Diluted plasma from serial draws of each subject were placed on the same ELISA plate. For longitudinal samples measured on different plates, samples from prior draws were rerun to calibrate the experimental variances across plates. For trajectory analysis, one phase decay model and linear regression model of log transformed antibody data were assessed in all cases (*24*). An extra sum-of-squares F Test was used for fitting curve comparisons. One phase decay model was used to report R, peak and plateau anti-S and anti-RBD IgG using log transformed data. Linear regression was used to report slope.

To test mAb binding to other coronavirus spike, ELISA plates were coated with 2 μg SARS spike, MERS spike, HKU1 spike, OC43 spike, NL63 spike or 229E spike in 30 μL PBS. To test mAb binding to SARS-CoV-2 subdomains, ELISA plates were coated with 4 μg SARS-CoV-2 S1, 2 μg SARS-CoV-2 NTD, 2 μg SARS-CoV-2 RBD, and 2 μg SARS-CoV-2 S2. Coated plates were incubated at 4°C overnight, and then blocked in 3% BSA as described above. Plates were then incubated with 1 μg/mL mAb in PBS containing 0.05% Tween20 at 4°C overnight. ELISA plates were washed and incubated with secondary antibody as described above. After plates were washed three times, ELISA plates coated with other coronavirus spike were incubated with 100 μL alkaline phosphatase substrate solution overnight, and ELISA plates coated with SARS-CoV-2 subdomains were incubated with substrate solution 2 hours. All plates were read at 405 nm through a microplate reader (Biotek Synergy H1). Binding signals were scored positive if OD_405_ values were above 5x PBS control on the same plate.

### mAb spike binding EC_50_ measurement

293T cells were co-transfected with pHDM-Wuhan-Hu-1 and pMAX-GFP plasmids at a 4:1 ratio by lipofectamine 3000 (Thermo fisher). 293T cell pool cotransfected with pHEF-VsVg and pMAX-GFP plasmids at a 4:1 ratio was used as a negative control. Cells were harvested 2 days post transfection and incubated with serially diluted mAbs. A total of nine three-fold serial dilutions were performed for each mAb with a starting concentration of 1-10 μg/mL. These dilutions were incubated with 2x10^5^ transfected 293T cells on ice for 20 min. Cells were washed and stained with DAPI and AF647-anti-human IgG (Thermo fisher, A-21445), and manually analyzed by flow cytometry (BD Canto II). GFP^+^ cells were gated to measure MFI (AF647 fluorescence) of S-expressing 293T cells. If MFI of 10 μg/mL mAb-treated pHDM-Wuhan-Hu-1/pMAX-GFP-co-transfected 293T cells at was at least 2 times greater than mAb treated negative control cells (pHEF-VsVg/pMAX-GFP co-transfected 293T cells), the mAb was scored as a spike binder. The data were acquired for each antibody from highest to lowest concentration. Data acquisition toward lower dilutions continued until mAb binding MFI in GFP^+^ cells achieved MFI values similar to negative controls. The average of MFI from 4 negative controls from each experiment was set as the background. The EC_50_ for each S-binding mAb was reported as the concentration with 50% of maximal binding determined by non-linear regression using Graphpad Prism. The EC_50_ for 3 out of 189 clones were set at the detection limit (10 μg/mL) due to poor regression curve fitting (<0.7). Raw data for EC50 values are included in Raw data file (supplementary data file 5).

### Activation induced markers (AIM) assay

After 2~3x10^6^ PBMCs were enriched for B cells through anti-CD19 magnetic beads (Miltenyi), flow through cells were centrifuged and incubated with 1 μg/mL peptide megapools consisting of SARS-CoV-2 spike, SARS-CoV-2 remainder (SARS-CoV-2 proteome minus spike), HCoVs (HKU1, OC43, NL63, 229E) spike, HCoVs remainder (HCoVs proteome minus spike), and cytomegalovirus (CMV), as well as phytohemagglutinin (500x, Thermo fisher) and DMSO. Cells were cultured in 96 well round-bottom plates with RPMI containing 10% FBS for 24 hours. Cells were stained with Biolegend antibodies FITC anti-CD27 (356404), PE anti-CCR7 (353204), Percp cy5.5 anti-CD4 (357414), PE-cy7 anti-CD8 (344712), APC anti-OX40 (350008), APC-Cy7 anti-CD45RA (304128), AF700 anti-CD3 (300424), BV510 anti-CD19 (302242), BV510 anti-CD14 (367124), BV605 anti-CD69 (310938), BV711 anti-CD137(309832).

### SARS-CoV-2 pseudovirus neutralization assay

293T cells were co-transfected with pHDM vector expressing SARS-CoV-2 variants, pLenti CMV Puro LUC (w168-1), and psPAX2 (Addgene) with lipofetamine 3000. Pseudoviruses in the supernatants were harvested two days after transfection and diluted in DMEM containing 10% FBS to test titers in 293T cells stably expressing human ACE2 and TMRPSS2 (provided by Dr. Marc Johnson, University of Missouri School of Medicine). For neutralization assays (*47*), plasma was serially diluted to incubate with pseudovirus at 37°C for 1 hour. The mixtures of diluted plasma and pseudovirus in duplicate were added to 293T cells stably expressing human ACE2 and TMRPSS2 (2x10^4^/well). Two days after the incubation, cells were lysed by Promega One-Glo luciferase reagent, and the luciferase signals were measured by Biotek Synergy H1. Plasma titers that achieved 50% neutralization (NT_50_) were determined by non-linear regression using Graphpad prism of log-transformed luciferase signal. For early neutralization response analysis, the second blood draw (instead of the first draw) of 9 COVID-19 convalescent subjects was used in neutralization test to Wuhan-Hu-1, D614G, Alpha, Beta, Gamma, Delta and Omicron variants because of the first month plasma samples were depleted in the antibody ELISA assays from these 9 subjects. This is why the median blood draw day is 38 for the convalescent antibody tests measured by ELISA (Fig. 1A and B) and 43 for the neutralization assays (Fig. 1D-J). The NT_50_ was set as the limited of detection (30) if the first plasma dilution (1:30) was less than 50% neutralization or if the regression fit was poor (< 0.7) (*18*). Twelve out of a total of 210 plasma samples (5.7%) showed an NT_50_ of 30 (detection limit) to the Wuhan-Hu-1 strain. NT_50_ data for these 12 samples to all variants are shown separately in Table S3 due to denominator 0 in the breadth index calculation.

### Statistical analysis

Statistical analysis was performed with Graphpad prism 8 software. After normality testing, log-transformed antibody titers were compared by student’s *t* test for two comparisons and ANOVA with Tukey’s test for multiple comparisons. Unless indicated, two tailed unpaired Mann-Whitney U tests were performed to analyze statistical differences between two comparisons; Two tailed Kruskal-Wallis test with Dunn’s multiple comparison correction was performed to analyze statistical differences in multiple comparisons. Spearman correlation was used for association analysis. Extra sum-of-squares F Test was used for comparisons of fitting curves. Unless indicated, horizontal lines in dot plots indicate median. The paired Wilcoxon test and mixed effect analysis were performed for comparison of pre-vaccination and post-vaccination comparisons in Cohort B. Analysis of Covariance (ANCOVA) was performed to test for significance of differences in slopes generated from simple linear regression. Statistical analysis was performed for all the comparisons as described in supplementary data S5. Asterisks were only shown for the statistical significant test (p value) less than 0.05.

## Supplementary Materials

### Other Supplementary Material for this manuscript includes the following:

MDAR Reproducibility Checklist
