# Supplementary material for: Immune recall improves antibody durability and breadth to SARS-CoV-2 variants
Source: Sci Immunol. 2022 May 12:eabp8328. doi: 10.1126/sciimmunol.abp8328 (PMC9097880; doi:10.1126/sciimmunol.abp8328)
Supplement: Supplementary file 1 — Figs. S1 to S8 Tables S1 to S4 [file sciimmunol.abp8328_sm.pdf]

Science Immunology
AAAS

Supplementary Materials for

Immune recall improves antibody durability and breadth to SARS-CoV-2 variants

Yuezhou Chen *et al.*

Corresponding author: Duane R. Wesemann, dwesemann@bwh.harvard.edu

DOI: 10.1126/sciimmunol.abp8328

The PDF file includes:

Figs. S1 to S8
Tables S1 to S4

Other Supplementary Material for this manuscript includes the following:

MDAR Reproducibility Checklist
Data files S1 to S5

Immune Recall Improves Antibody Durability and Breadth to SARS-CoV-2 Variants

Yuezhou Chen, Pei Tong, Noah Whiteman, Ali Sanjari Moghaddam, Mehrdad Zarghami, Adam Zuiani, Shaghayegh Habibi, Avneesh Gautam, Keerti, Caihong Bi, Tianshu Xiao, Yongfei Cai, Bing Chen, Donna Neuberg, Duane R. Wesemann

Supplementary materials	Page
Figure S1. Sample collection timeline and cohort characteristics	2-3
Figure S2. Antibody response comparisons between Moderna and Pfizer-BioNTech vaccinees	4-5
Figure S3. Cross-variant neutralization level and breadth index in COVID-19 convalescents before and after vaccination	6-7
Figure S4. T cell analysis in sustainers versus decayers after natural infection	8-9
Figure S5. S^+ memory B cell sorting strategy, characteristics of mAbs and plasma cross-reactivity analysis	10-11
Figure S6. S^+ memory B cell repertoire and clonal relationship analysis	12-13
Figure S7. Analysis of neutralization function in sustainers versus decayers after natural infection	14
Figure S8. Anti-SARS-CoV-2 antibody and neutralization level in sustainers and decayers after vaccination	15
Table S1: Cohort characteristics	16-17
Table S2: Anti-SARS-CoV-2 antibody trajectory parameters from one-phase decay and linear regression	18
Table S3: Cross-variant NT_{50} values for the samples with neutralization to Wuhan-Hu-1 strain at limit of detection	19
Table S4: Process of heavy and light chain sequences for Spike-binding mAb production	20

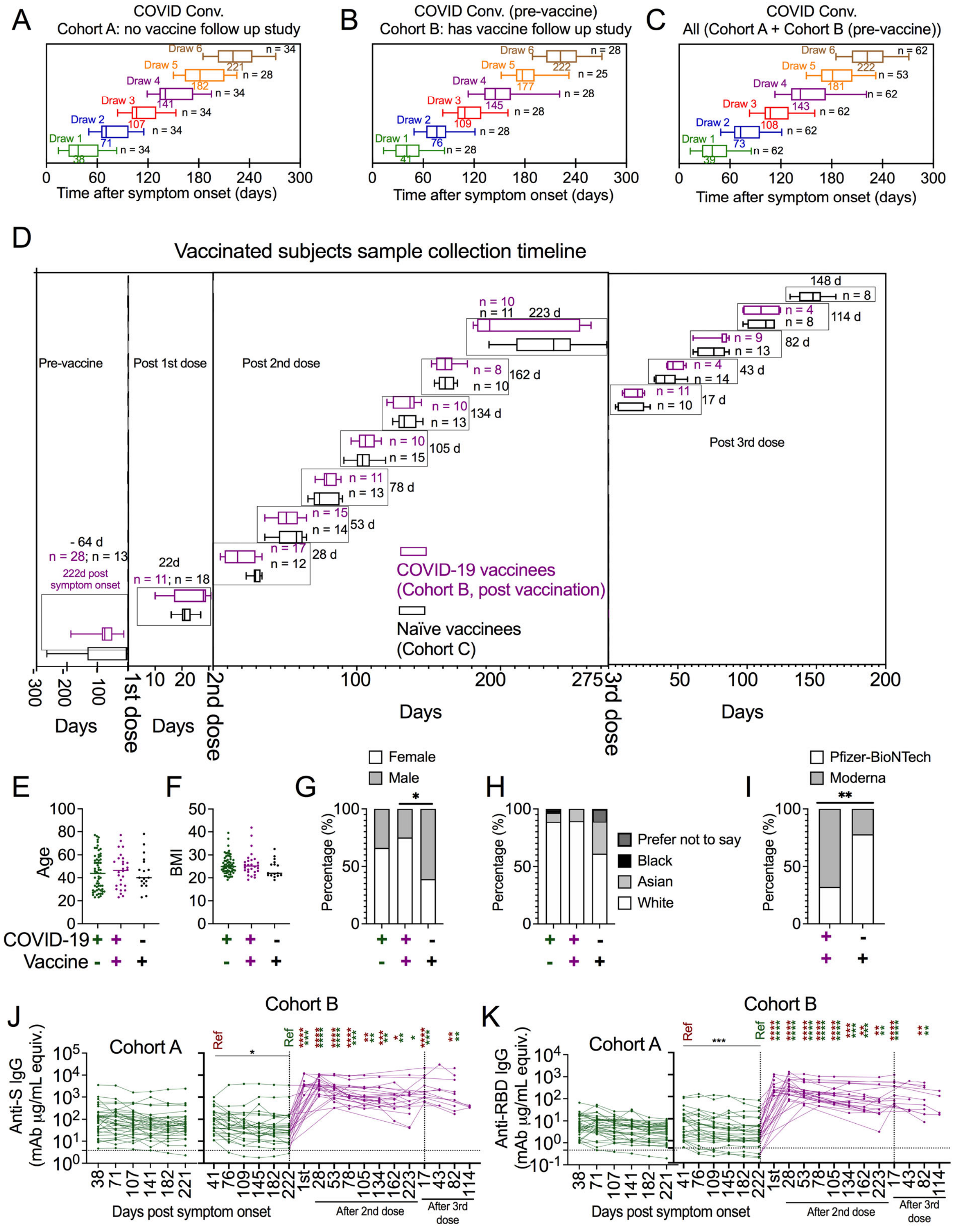

Figure S1. Sample collection timeline and cohort characteristics. (A-C) Box and whisker plots showing blood collection timeline for seroconverted COVID-19 convalescent subjects in

cohort A (without vaccination follow up, n = 34), cohort B (with vaccination follow up, n = 28) (pre-vaccination) and the combination of cohort A and cohort B (pre-vaccination) (n = 62). Median time is indicated. (D) Box and whisker plots showing blood collection timeline for COVID-19 vaccinees (purple, n = 28, cohort B, post vaccination) and naïve subjects (black, n = 18, cohort C) with mRNA vaccination. The x-axis shows days before 1st dose, after 1st dose, 2nd dose and 3rd dose. Median days and number of subjects donating blood in each time interval are indicated. (E-H) Dot and bar graphs of age (survey 100% complete), BMI (survey 94% complete), gender (survey 100% complete) and race (survey 89% complete) distributions in COVID-19 convalescent subjects before vaccination, COVID-19 vaccinees, and naïve vaccines as described in (A-D). Kruskal-Wallis test was performed for E and F. Fisher exact test was performed for G. (I) Bar graph showing vaccine type distribution in COVID-19 vaccinees, and naive vaccines as described in (D). Fisher exact test. (J, K) Dot plots showing anti-S (J) and anti-RBD (K) IgG antibody levels in seroconverted COVID-19 convalescents cohort A (green, n = 34, left) and cohort B (n = 28) before (green) and after (purple) vaccination over time as indicated. After the log-transformation of the antibody data, mixed-effects analysis was performed within cohort B. *p<0.05, **p<0.01, ***p<0.001, ****p<0.0001.

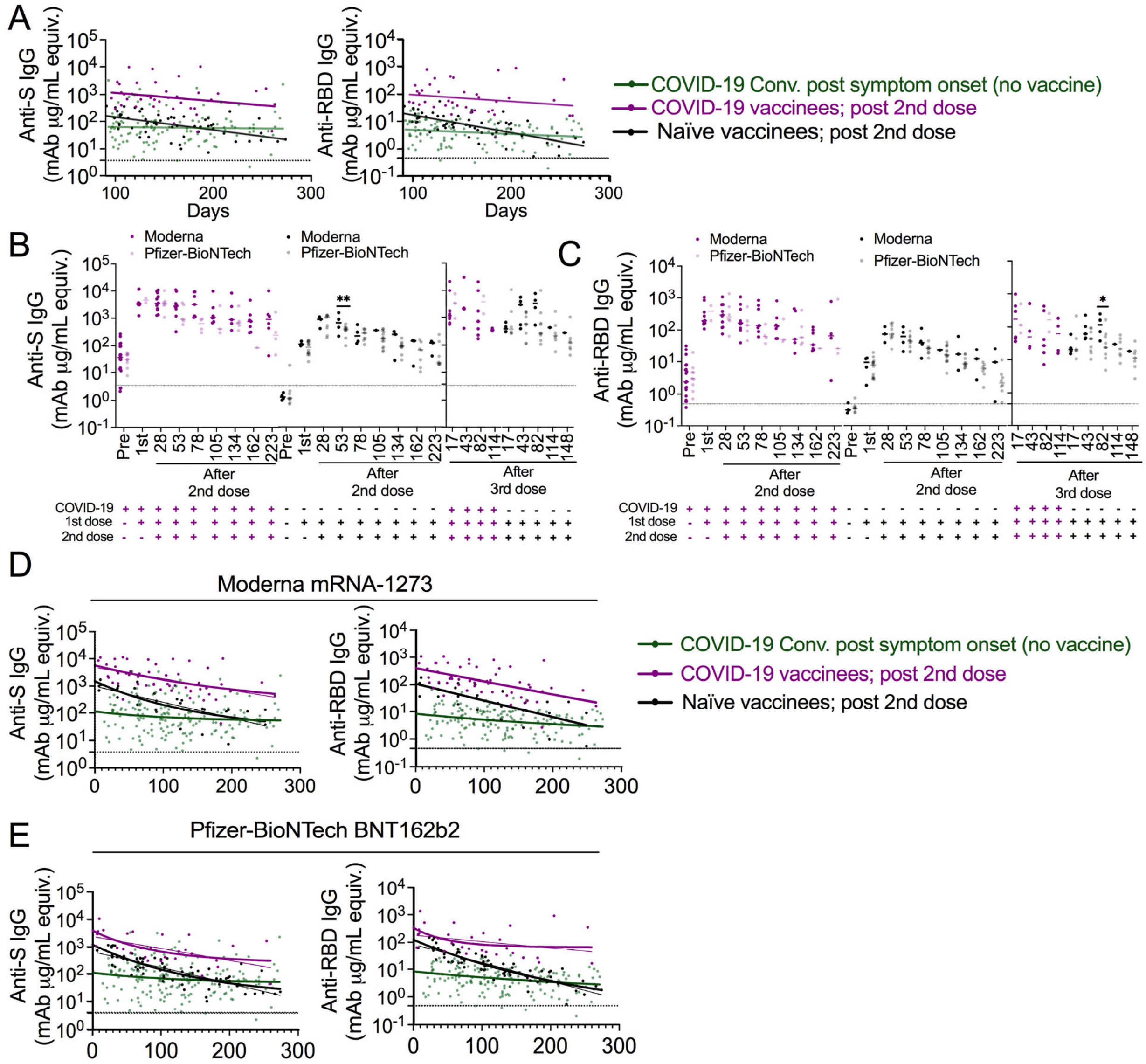

Figure S2. Antibody response comparisons between Moderna and Pfizer-BioNTech vaccinees. (A) Dot and line graph showing anti-S IgG (left) and anti-RBD IgG (right) linear regression in COVID-19 convalescents (green, n = 34) after natural infection, COVID-19 vaccinees (purple, n = 28) and naïve vaccinees (black, n = 18) after 2nd dose. The first 90-days data were excluded for all groups. (B, C) Dot plots showing plasma anti-S (B) and anti-RBD (C) IgG level in COVID-19 vaccinees (purple, n = 4-19) and naïve vaccinees (black, n = 1-5) receiving Moderna mRNA-1273 vaccination, and COVID-19 vaccinees (light purple, n = 1-9) and naïve vaccinees (grey, n = 6-14) receiving Pfizer-BioNTech BNT162b2 vaccination at the indicated median collection time point (days). 2-way ANOVA. (D, E) Dot and line graph showing anti-S (left) and anti-RBD (right) IgG trajectory over time with one phase decay model (thick line) and linear regression (thin line) fitted curves in COVID-19 convalescents (green), as well as

COVID-19 vaccinees (purple) and naïve vaccinees (black) receiving Moderna mRNA - 1273 vaccination (D), and Pfizer-BioNTech BNT162b2 mRNA vaccination (E). *p<0.05, **p<0.01.

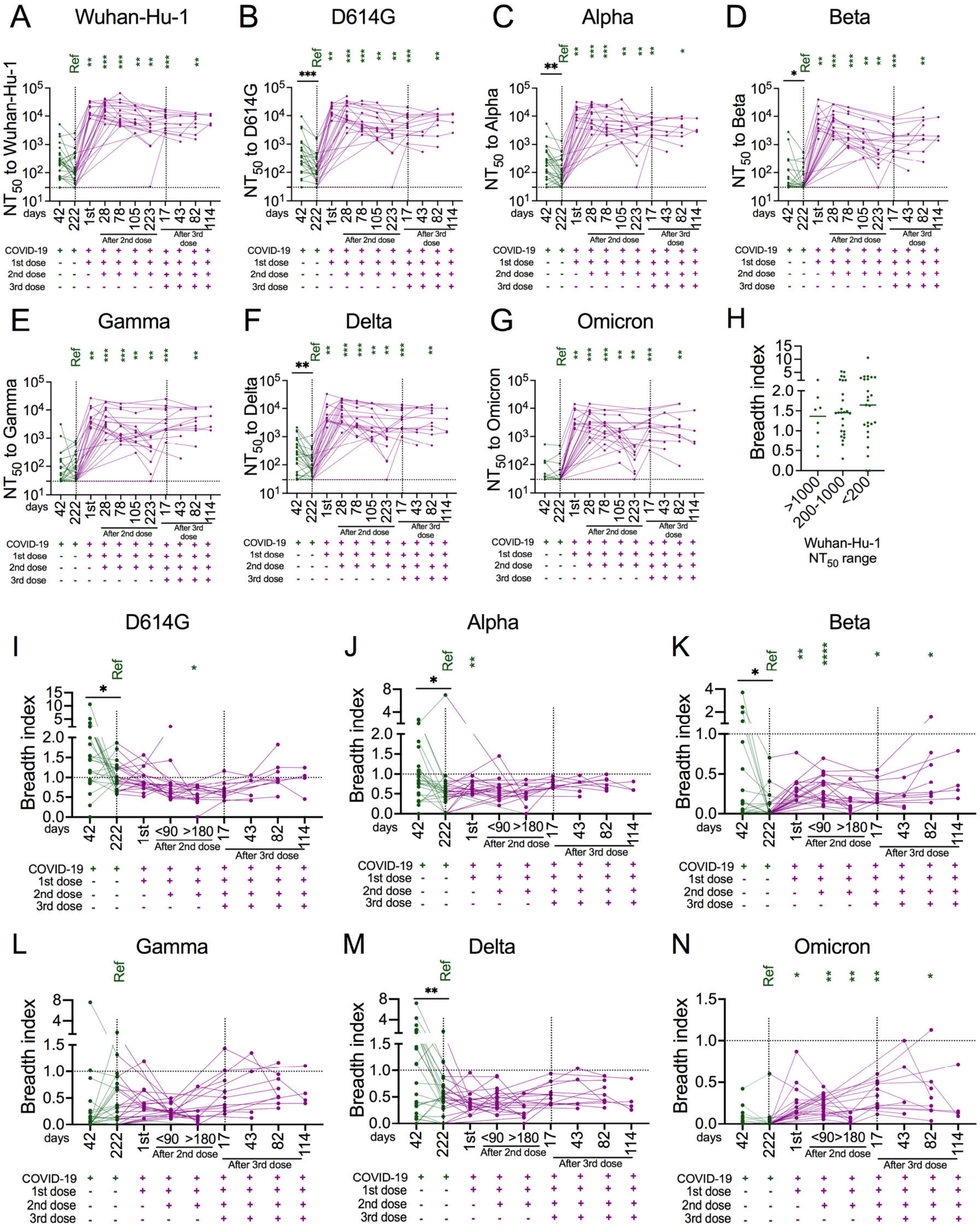

Figure S3. Cross-variant neutralization level and breadth index in COVID-19 convalescents before and after vaccination. (A-G) Dot and line graph illustrating pseudovirus NT_{50} to indicated variants in plasma of COVID-19 convalescents (cohort B) before (green) (n = 28) and after (purple) (n = 3-14) the vaccination over the time as indicated. Wilcoxon test. (H) Breadth index in binned groups of COVID-19 convalescents before vaccination (42 days post

symptom onset) based on NT_{50} to Wuhan-Hu-1 strain as >1000 (n = 8), 200-1000 (n = 26), and <200 (n = 25). Kruskal-Wallis test. (I-N) Dot and line graphs illustrating breadth index to indicated variants in plasma of COVID-19 convalescents before (green) (n = 24-25) and after (purple) (n = 3-21) the vaccination over the time as indicated. Wilcoxon test. *p<0.05, **p<0.01, ***p<0.001; ****p<0.0001.

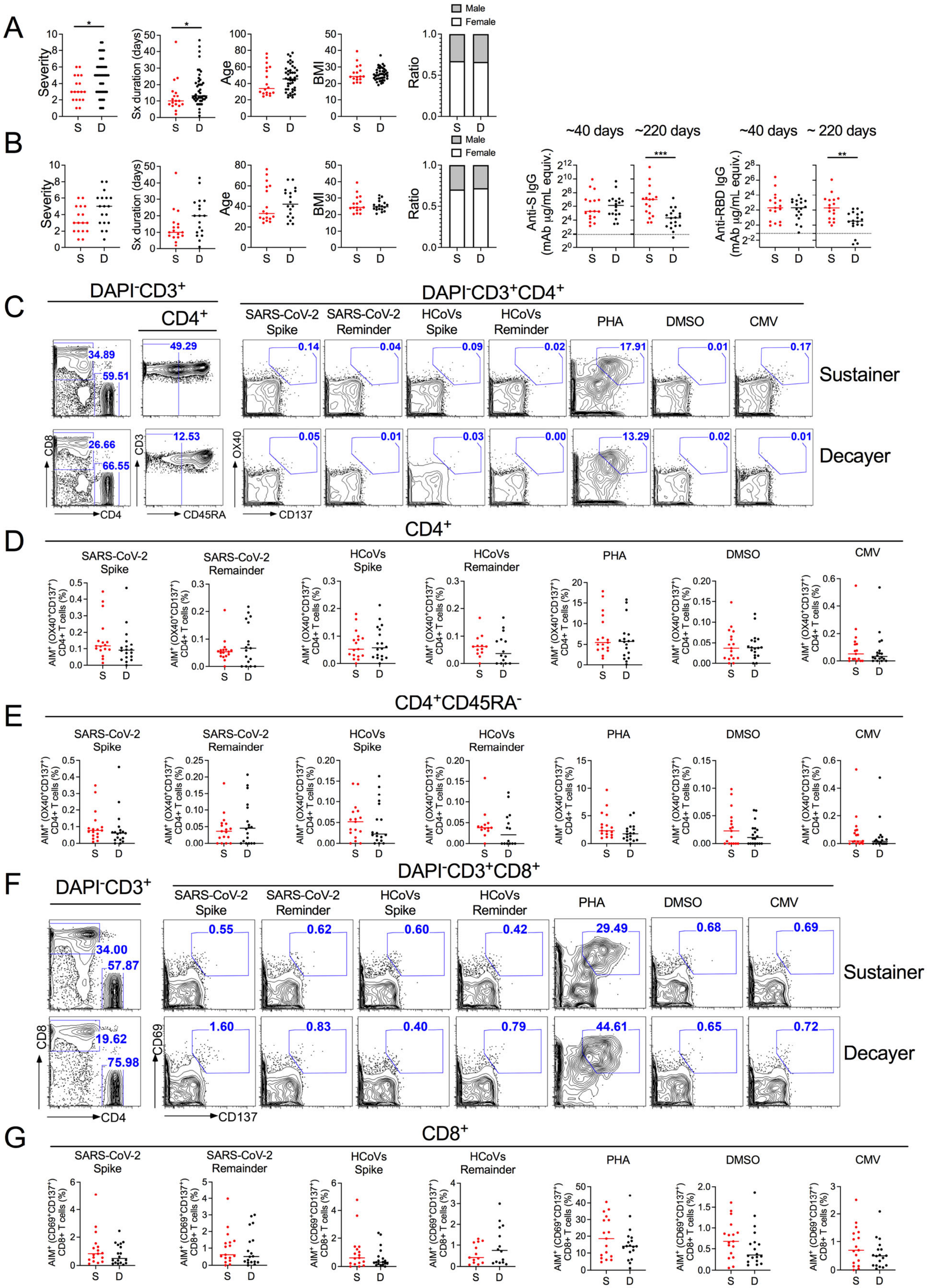
A
B
C
D
E
F
G
Severity
Sx duration (days)
Age
BMI
Ratio
Male
Female
S
D
~40 days
~220 days
Anti-S IgG (mAb μg/mL equiv.)
Anti-RBD IgG (mAb μg/mL equiv.)
DAPI-CD3+
CD4+
DAPI-CD3+CD4+
SARS-CoV-2 Spike
SARS-CoV-2 Reminder
HCoVs Spike
HCoVs Reminder
PHA
DMSO
CMV
Sustainer
Decayer
CD8
CD4
CD3
CD45RA
OX40
CD137
CD4+CD45RA-
SARS-CoV-2 Remainder
HCoVs Remainder
AIM+ (OX40+CD137+) CD4+ T cells (%)
DAPI-CD3+CD8+
CD69
CD8+
AIM+ (CD69+CD137+) CD8+ T cells (%)

Figure S4. T cell analysis in sustainers versus decayers after natural infection. (A) Plots showing the distributions of symptom duration, severity, age, BMI, and genders between sustainers (red, n = 18) and decayers (black, n = 44). Surveys were more than 94% complete for each category. Mann-Whitney U test. (B) Plots showing the distributions of symptom duration, severity, age, BMI, genders, anti-S IgG and anti-RBD IgG between sustainers (red, n = 17) and decayers (black, n = 18) selected for the T cell analysis. Surveys were more than 94% complete for each category. Mann-Whitney U test. Unpaired t test for log-transformed antibody data. (C-G) Representative flow cytometry plots (C, F) and dot plots (D, E, G) showing the gating strategy and percentage of $AIM^+(OX40^+CD137^+)$ cells (C, D, E) and AIM^+ $(CD69^+CD137^+)$ cells (F, G) gated on live $CD4^+$ (D), $CD4^+CD45RA^-$ (E), and $CD8^+$ (F) cells as indicated in sustainers (n = 13-17) and decayers (n = 14-18) after day ~40 post-symptom-onset PBMCs were stimulated with SARS-CoV-2 and seasonal HCoVs peptide megapools consisting of full length spike or non-spike peptides remaining, termed remainders (i.e., whole proteome minus spike). Mann-Whitney U test. *p<0.05, **p<0.01, ***p<0.001.

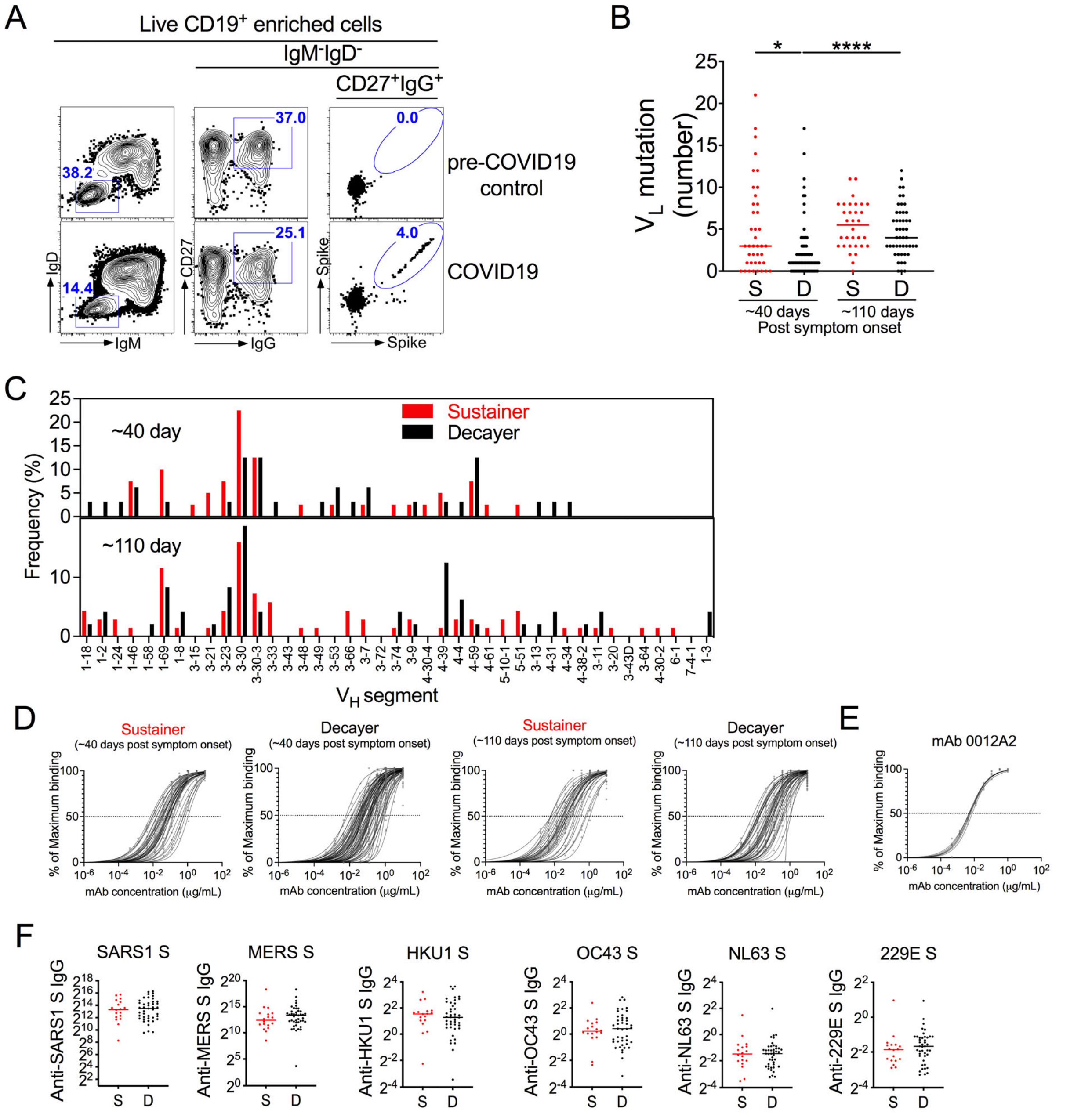

Figure S5. S^+ memory B cell sorting strategy, characteristics of mAbs and plasma cross-reactivity analysis. (A) Representative flow cytometry plot showing the gating strategy to sort S^+ memory B cells. (B) Dot plot showing mAb light chain V gene segment (V_H) mutation number per sequence cloned from S^+ memory B cells in sustainer (S, red) and decayer (D, black) collected ~ 40 days after symptom onset (sustainer, n = 9, 40 clones; decayer, n = 11, 69 clones;1st draw) and ~110 days after symptom onset (sustainer, n = 8, 32 clones; decayer, n = 9, 48 clones; 3rd draw). Kruskal-Wallis test. (C) Bar graph showing the frequency of V_H gene segment usage in the clones as describe in (B). (D) Regression curves showing the percent of

maximum binding of serial diluted mAbs to spike expressed on 293T cells. mAbs were isolated from sustainers (40 clones) and decayers (67 clones) ~40 days post symptom onset, as well as sustainers (32 clones) and decayers (47 clones) ~110 days post symptom onset. EC_{50} were set at the detection limit (10 μg/mL) for 3 of the 189 clones that had poor regression curve fitting (<0.7). (E) Regression curves showing the percent of maximum binding of serial diluted mAb C12A2 to spike expressed on 293T cells as a reference in 7 independent experiments to test the robustness of the EC_{50} measurement assay. (F) Dot plots showing HCoV spike-specific IgG in plasma collected in sustainers (n = 18) and decayers (n = 43) ~40 days after symptom onset. Unpaired t test for log-transformed antibody data. *p<0.05, ****p<0.0001.

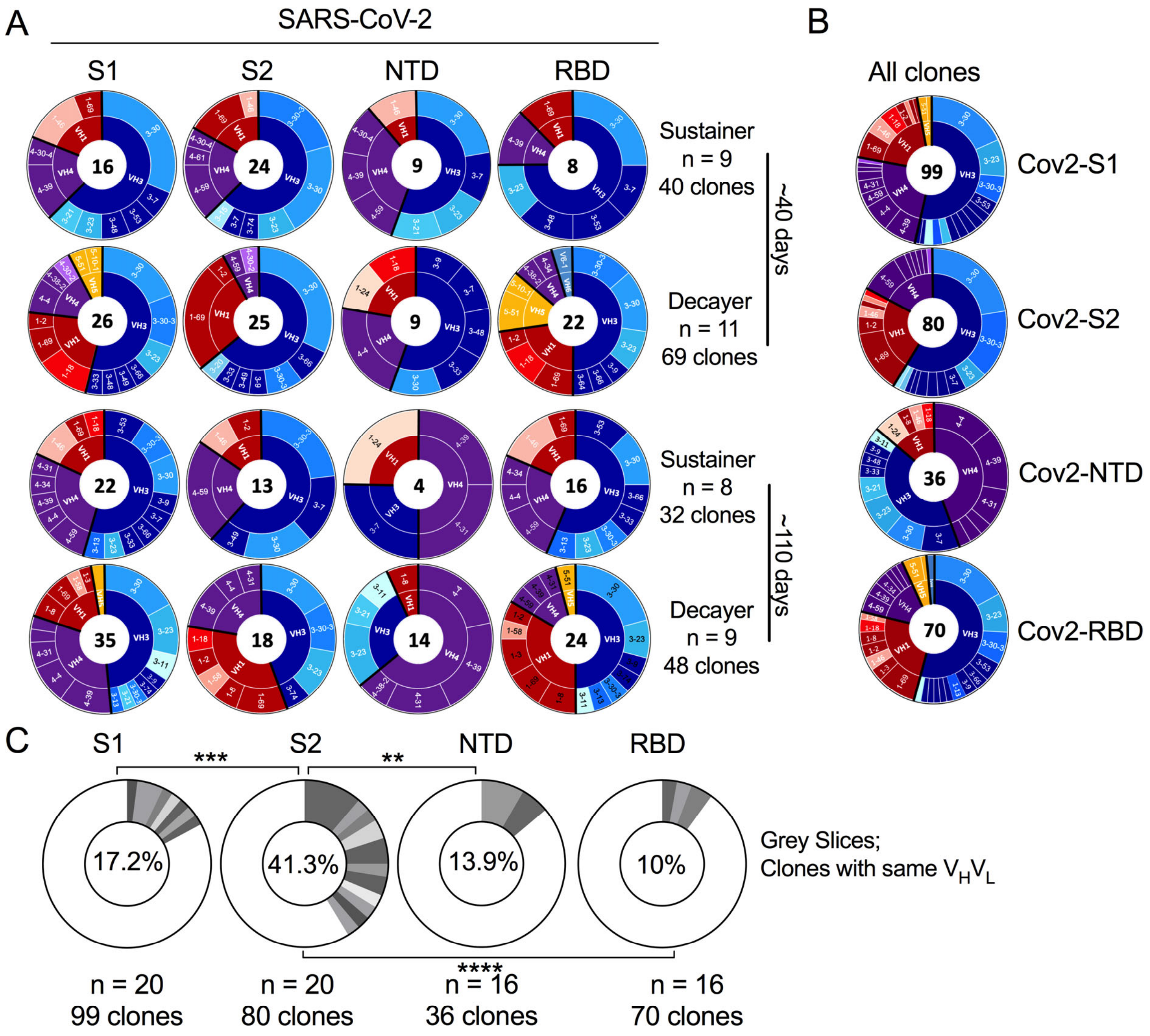

D

Cross-reactive clones from early S⁺ memory B cells

			Heavy				Light							
Clone ID	Donor ID	Source	IGHV	IGHD	IGHJ	CDRH3	IGLV	IGLJ	CDRL3	SARS1 S	MERS S	HKU1 S	OC43 S	CoV2 S2
492_P1_A5	820#0089	S	IGHV3-30-3*01	IGHD2-21*01	IGHJ3*02	ARGGGGGDGAFDN	IGKV2-30*01	IGKJ2*01	MQTTHWPYT	+	-	+	+	+
0007A2	820#0007	S	IGHV3-23*01	IGHD2-15*01	IGHJ6*02	AKVRGYCSGGRGCMDV	IGKV1-13*02	IGKJ4*01	QQFNTYPALT	+	+	+	+	+
495_P1_A5	820#0007	S	IGHV3-23*01	IGHD2-15*01	IGHJ6*02	....	IGKV1-13*02	IGKJ4*01	....	+	+	+	+	+
494_P1_A6	820#0020	S	IGHV3-74*01	IGHD6-13*01	IGHJ6*02	ARVAVVHLRSSWYFYGLDV	IGKV1-6*01	IGKJ1*01	LQDYIYPPT	-	+	+	+	+
0007A9	820#0007	S	IGHV4-59*01	IGHD2-15*01	IGHJ4*02	ARETRWNYVDY	IGKV4-1*01	IGKJ1*01	QQYHGTPWT	+	+	+	+	+
492_P1_A8	820#0089	S	IGHV4-59*01	N/A	IGHJ5*02	....PS.WL.R	IGKV4-1*01	IGKJ1*01	H..FD....	+	+	+	+	+
0015C3	820#0015	D	IGHV4-59*01	N/A	IGHJ5*02	.....Y.WF.P	IGKV4-1*01	IGKJ1*01	...FT-....	-	+	+	+	+
0007A4	820#0007	S	IGHV3-30*03	IGHD6-13*01	IGHJ4*02	ARAGFSSSWALGDYFDY	IGKV4-1*01	IGKJ2*01	QQYYSPLPYT	+	+	+	+	+
496_P1_D5	820#0310	S	IGHV3-7*03	IGHD3-22*01	IGHJ5*02	ARDSYYYDSSGFGVNWFDP	IGLV1-40*01	IGLJ1*01	QSYDSSLSGYV	+	+	+	+	+
494_P1_B8	820#0020	S	IGHV1-46*03	IGHD3-10*01	IGHJ1*01	ARGAITMVRGAVESAEYFQH	IGKV4-1*01	IGKJ4*01	QQYYNTPLT	+	-	+	+	+
0007A8	820#0007	S	IGHV3-30*18	IGHD3-3*01	IGHJ4*02	AKGSRFLEWLSEY	IGKV1-17*03	IGKJ1*01	LQHNSYPWT	+	+	+	+	+
0018B1	820#0018	D	IGHV1-69*09	IGHD5-24*01	IGHJ4*02	ARALGGDGYNKPHYFDH	IGKV1-39*01	IGKJ1*01	QQSYNAPWT	+	+	+	+	+
0018C8	820#0018	D	IGHV3-49*04	IGHD1-14*01	IGHJ4*02	TRGLKVEPYYFDY	IGKV1-5*03	IGKJ2*01	QQYNSYLYT	+	-	+	+	+
0012HE9	820#0012	D	IGHV1-69*01	IGHD2-21*02	IGHJ4*02	ARGSVGLGGDCYQCGYYFDY	IGKV1-5*03	IGKJ1*01	QQYNSYSRG	+	-	+	+	-
0012B3	820#0012	D	IGHV3-20*04	IGHD2-2*01	IGHJ6*03	ARVLVPAAYPYYYYYMDV	IGLV3-9*01	IGLJ2*01	QVWDSSTAHVV	+	+	+	-	+
0018C11	820#0018	D	IGHV3-30-3*01	IGHD3-10*01	IGHJ4*02	ASLITMVRGVFDY	IGKV1-39*01	IGKJ4*01	QQSYSTPLT	-	-	+	+	+

Figure S6. S⁺ memory B cell repertoire and clonal relationship analysis. (A, B) Donut plots showing the V_H gene segment usage of the mAb isolated from S⁺ memory B cells categorized

by their binding to SARS-CoV-2 spike subdomains in sustainers and decayers ~40 or ~110 days post symptom onset (A) or all subjects at all time points (B). The number in the inner circle indicates number of clones in the indicated category. (C) Donut plots showing the distribution of mAb sequences isolated from all subjects at all time points categorized by their binding to SARS-CoV-2 subdomains with the number of clones and subjects listed below each donut. Each Grey slice shows the clones with the same V_H and V_L gene segments, and white indicates the clones harboring unique V_H and V_L gene segment combinations. The number in the inner circle shows the percentage of clones with clonal relationship added together. Fisher exact test. (D) Sequence alignment of cross-reactive mAb isolated from sustainers (n = 4, 10 clones) and decayers (n = 3, 6 clones) ~40 days post symptom onset. **p<0.01, ***p<0.001.

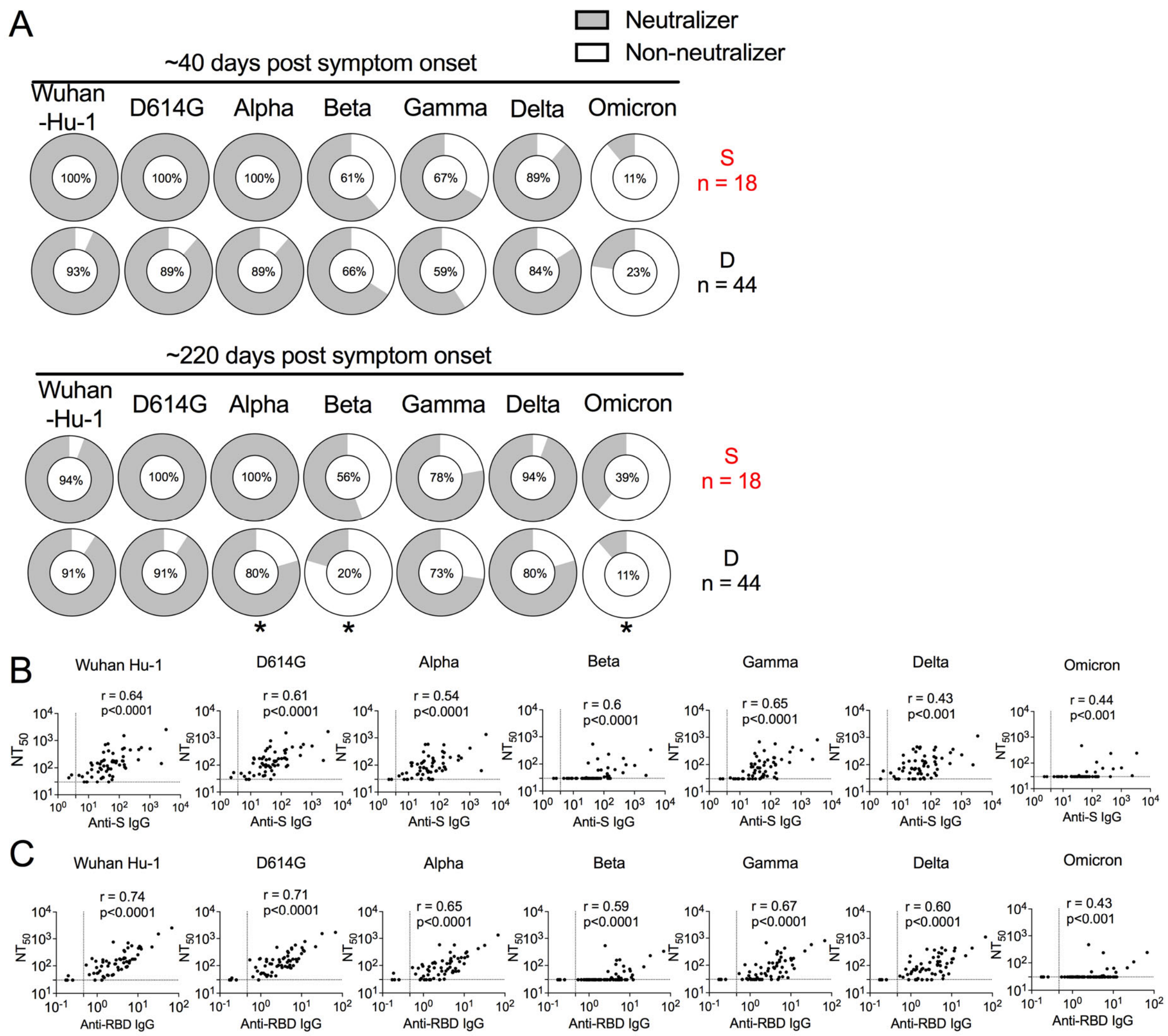

Figure S7. Analysis of neutralization function in sustainers versus decayers after natural infection. (A) Donut plots illustrating the percentage of neutralizers harboring neutralization titer > 30 (detection limit) to the indicated pseudotyped variants in sustainers (n = 18) and decayers (n = 44) ~40 days (left) and ~220 days (right) after symptom onset. Fisher exact test. (B, C) Scatter plots illustrating Spearman correlation between anti-S (B) and anti-RBD (C) IgG in plasma isolated ~220 days after symptom onset from COVID-19 convalescents (n = 62) and neutralization titers to the indicated variants. *p<0.05, **p<0.01, ***p<0.001, ****p<0.0001.

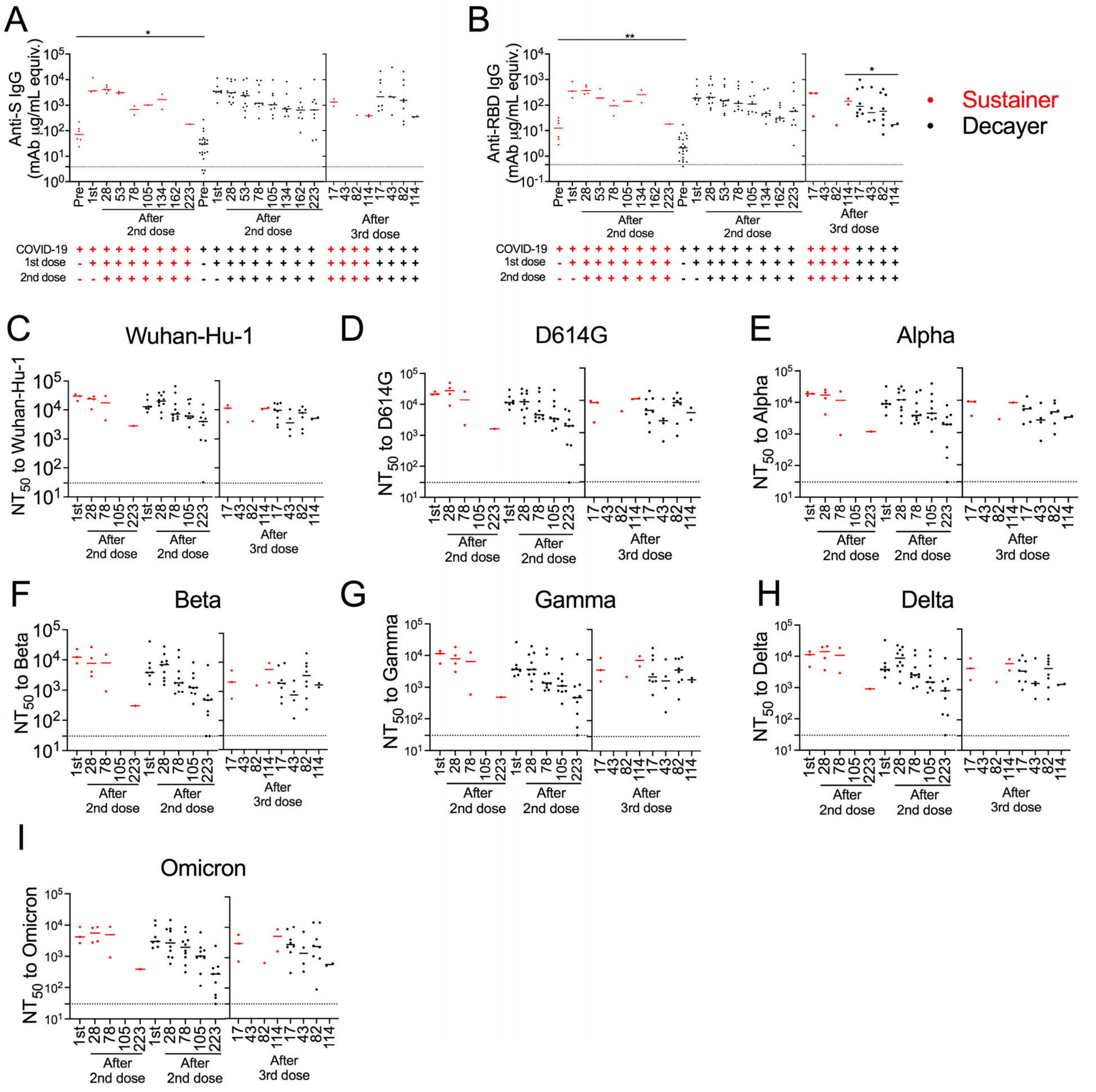

Figure S8. Anti-SARS-CoV-2 antibody and neutralization level in sustainers and decayers after vaccination. (A, B) Anti-S (A) and anti-RBD (B) IgG antibody levels in sustainer (red, n = 1-6) and decayers (black, n = 2-22) before and after vaccination over time as indicated. Unpaired t-test was performed to test the significant difference of log-transformed antibody data. The dashed lines represent twice of the average of pre-COVID-19 controls. (C – I) Dot plots showing 50% pseudovirus neutralization titers (NT_{50}) to indicated SARS-CoV-2 variants in plasma from sustainers (red, n = 1-4) and decayers (black, n = 2-10) after vaccination over time as indicated. The Dashed lines represent the limit of neutralization detection (i. e. 30). Mann-Whitney U test. *p<0.05, **p<0.01.

Table S1: Cohort characteristics

	**Cohort A**	**Cohort B**	**Cohort A + Cohort B (pre-vaccination)**	**Cohort C**
	n=34	n= 28	n=62	n=18
**Demographics**				
Median Age (years)	42.7 (range 24-75)	46.4 (range 23-77)	44.9 (range 23-77)	39.8 (range 22-77)
Sex				
Men	14	7 (25%)	21 (33.9%)	11 (61.1%)
Women	20	21 (75%)	41 (66.1%)	7 (38.9%)
Race				
White	30 (88.2%)	25 (88.3%)	55 (88.7%)	11 (61.1%)
Black/ African American	1 (2.9%)	0 (0.0%)	1 (1.6%)	0 (0.0%)
Asian	2 (5.9%)	3 (10.7%)	5 (8.1%)	5 (27.8%)
Other	0 (0.0%)	0 (0.0%)	0 (0.0%)	0 (0.0%)
Prefer not to say	1 (2.9%)	0 (0.0%)	1 (1.6%)	2 (11.1%)
Hispanic or Latino	1 (2.9%)	1 (3.6%)	2 (3.0%)	4 (22.2%)
**General Characteristics**				
Number of Household Members (median)	2 (range 1-6)	2 (range 1-5)	2 (range 1-6)	4 (range 2-6)
Healthcare worker	15 (44.11%)	21 (75%)	36 (58.1%)	12 (66.67%)
**Clinical Characteristics**				
Median BMI	24.3 (range 20.1-39.5)	25.2 (range 19.2-41.8)	25.0 (range 19.2-39.5)	22.1 (range 19.1-32.6)
Lung Disease	4 (11.7%)	3 (10.7%)	7 (11.3%)	1 (5.6%)
Diabetes	0 (0.0%)	0 (0.00%)	0 (0.0%)	1 (5.6%)
Cardiovascular Disease	4 (11.7%)	2 (7.1%)	6 (9.7%)	1 (5.6%)
Immunocompromised	0 (0.0%)	2 (3.6%)	2 (3.2%)	0 (0.00%)
**COVID-19 Symptoms**				
Fever	22 (42.9%)	12 (42.9%)	34 (54.8%)	N/A
Cough	24 (70.6%)	17 (60.7%)	41 (66.1%)	N/A
Shortness of Breath	16 (47.1%)	11 (39.3%)	27 (43.6%)	N/A
Sore Throat	13 (38.24%)	10 (35.7%)	23 (37.1%)	N/A
Headaches	24 (70.6%)	17 (60.7%)	41 (66.1%)	N/A
Body Aches	27 (79.4%)	20 (71.4%)	47 (75.8%)	N/A

Loss of smell or taste	24 (70.6%)	20 (71.4%)	44 (71.0%)	N/A
Congestion	21 (61.8%)	13 (46.4%)	34 (54.8%)	N/A
Nausea	7 (20.6%)	8 (28.6%)	15 (24.2%)	N/A
Diarrhea	8 (23.53%)	11 (39.3%)	19 (30.7%)	N/A
Hospitalization	0 (0.0%)	2 (7.4%)	2 (3.2%)	N/A
Duration of Symptoms (median)	12 (range 2-47)	20 (range 1-46)	12.5 (range 1-47)	N/A
Severity of Symptoms (median)	5 (range 1-9)	3.5 (range 1-9)	4.5 (range 1-9)	N/A
**mRNA vaccination**				
Vaccine brand				
Pfizer-BioNTech		9 (32.1%)		14 (77.8%)
Moderna		19 (67.9%)		4 (22.2%)
Blood draw intervals				
Symptom onset date to pre-vaccination visit		222 (range 189-271)		N/A
Pre-vaccination visit to 1st vaccination		76 (range 13-187)		5 (range 1-266)
1st vaccination to post-1st vaccination visit		28 (range 10-31)		21 (range 16-27)
1st vaccination to 2nd vaccination		28 (range 21-43)		21 (range 20-43)
2nd vaccination to first post 2nd vaccination visit		17 (range 5-34)		30 (range 23-34)
3rd vaccination to first post 3rd vaccination visit		21 (range 10-26)		7 (range 5-30)

Table S2: Anti-SARS-CoV-2 antibody trajectory parameters from one-phase decay and linear regression

Type	Groups	All data				First 3-months data excluded
		One phase decay model			Linear regression	Linear regression
		R	Peak	Plateau	Slope	Slope
Anti-S IgG	COVID-19 Conv.	-0.13	2.05	1.691	-0.00102	-0.00036
	COVID-19 vaccinees	-0.52	3.669	2.145	-0.0043	-0.00302
	Naïve vaccinees	-0.8	3.112	0.951	-0.0062	-0.00473
Anti-RBD IgG	COVID-19 Conv.	-0.25	0.92	0.068	-0.001686	-0.00148
	COVID-19 vaccinees	-0.48	2.57	1.375	-0.00376	-0.00243
	Naïve vaccinees	-0.85	2.047	-3.612	-0.00704	-0.00643

Table S3: Cross-variant NT_{50} values for the samples with neutralization to Wuhan-Hu-1 strain at limit of detection.

	Neutralization NT_{50}							
Sample ID	Wuhan-Hu-1	D614G	Alpha	Beta	Gamma	Delta	Omicron	Cohort
820#0007-6	30	46.47	44.95	30	30	30	30.59	A
820#0225-1	30	30	30	30	30	30	30	B (pre-vaccination)
820#0231-1	30	30	30	30	30	30	30	B (pre-vaccination)
820#0453-1	30	30	30	35.95	30	30	30	B (pre-vaccination)
820#0225-6	30	30	33.01	30	30	30	30	B (pre-vaccination)
820#0231-6	30	30	30	30	30	30	30	B (pre-vaccination)
820#0453-6	30	30	51.76	30	30	30	30	B (pre-vaccination)
820#0857-6	30	30	30	30	30	30	30	B (pre-vaccination)
837V0033-1	30	30	30	30	30	30	30	C
837V0505-2	30	30	30	30	30	30	30	C

Table S4: Process of heavy and light chain sequences for Spike-binding mAb production

			Single cell PCR		Paired IgH and IgL clones		Spike binding mAbs	
			Sustainer	Decayer	Sustainer	Decayer	Sustainer	Decayer
All subjects	Draw 1	Heavy	93 (n = 10)	143 (n = 12)	53 (n = 9)	78 (n = 11)	40 (n = 9)	69 (n = 11)
		Light	93 (n = 12)	136 (n = 11)				
	Draw 3	Heavy	80 (n = 9)	124 (n = 10)	42 (n = 9)	64 (n = 10)	32 (n = 8)	48 (n = 9)
		Light	89 (n = 10)	113 (n = 10)				
Subject 820#0007	Draw 1	Heavy	9	NA	8	NA	5	NA
		Light	8	NA				
	Draw 3	Heavy	6	NA	2	NA	1	NA
		Light	3	NA				
